# FTIR and Raman Spectroscopy-Based Biochemical Profiling Reflects Genomic Diversity of Clinical *Candida* Isolates That May Be Useful for Diagnosis and Targeted Therapy of Candidiasis

**DOI:** 10.3390/ijms20040988

**Published:** 2019-02-25

**Authors:** Leszek Potocki, Joanna Depciuch, Ewelina Kuna, Mariusz Worek, Anna Lewinska, Maciej Wnuk

**Affiliations:** 1 Department of Genetics, Faculty of Biotechnology, University of Rzeszow, Pigonia 1, 35-310 Rzeszow, Poland; lpotok@o2.pl (L.P.); gawel.ewelina@gmail.com (E.K.); 2 Institute of Nuclear Physics, Polish Academy of Sciences, 31-342 Krakow, Poland; joannadepciuch@gmail.com; 3 Department of Microbiology, Faculty of Medicine, University of Rzeszow, 35-959 Rzeszow, Poland; mariusz.worek@gmail.com; 4 Department of Cell Biochemistry, Faculty of Biotechnology, University of Rzeszow, 35-310 Rzeszow, Poland

**Keywords:** *Candida albicans*, FTIR and Raman spectroscopy, karyotyping, genomic diversity, candidiasis

## Abstract

Despite the fact that *Candida albicans* is documented to be the main cause of human candidiasis, non-*C. albicans Candida* (NCAC) species, such as *Candida glabrata* and *Candida tropicalis*, are also suggested to be implicated in the etiopathogenesis of opportunistic fungal infections. As biology, epidemiology, pathogenicity, and antifungal resistance of NCAC species may be affected as a result of genomic diversity and plasticity, rapid and unambiguous identification of *Candida* species in clinical samples is essential for proper diagnosis and therapy. In the present study, 25 clinical isolates of *C. albicans*, *C. glabrata*, and *C. tropicalis* species were characterized in terms of their karyotype patterns, DNA content, and biochemical features. Fourier transform infrared (FTIR) spectra- and Raman spectra-based molecular fingerprints corresponded to the diversity of chromosomal traits and DNA levels that provided correct species identification. Moreover, Raman spectroscopy was documented to be useful for the evaluation of ergosterol content that may be associated with azole resistance. Taken together, we found that vibrational spectroscopy-based biochemical profiling reflects the variability of chromosome patterns and DNA content of clinical *Candida* species isolates and may facilitate the diagnosis and targeted therapy of candidiasis.

## 1. Introduction

Fungal infections caused by *Candida* species are either mucosal or systemic, in which the fungus invades and penetrates internal organs or tissues and/or reaches the bloodstream and spreads throughout the body (candidemia) [1,2]. Invasive candidiasis, which accounts for approximately three-fourths of systemic fungal infections, may be a life threatening condition, especially in a case of immunocompromised patients [2,3,4]. The *Candida* genus is composed of more than 150 heterogeneous species, but just a few of them have been implicated in human candidiasis [1]. While *C. albicans* causes the majority of human infections [1], the number of fungal infections caused by non-*C. albicans Candida* (NCAC) species, such as *C. glabrata*, *C. parapsilosis*, and *C. tropicalis* has been significantly elevated [5,6,7]. This may be due, at least in part, to improvements in diagnostic procedures, such as the use of chromogenic media with the ability to differentiate *Candida* species and some molecular diagnostic techniques, e.g., PCR [8]. In addition, some of the NCAC species associated with candidiasis are also less susceptible to antifungal drug treatment compared to *C. albicans* [9,10]. The biology, epidemiology, pathogenicity, and the mechanisms of antifungal resistance of NCAC species have not been studied as extensively as those of *C. albicans* [11].

Despite the fact that the techniques of isolation and identification of *Candida* species have been improved [11,12,13,14], the laboratory diagnosis of candidiasis remains challenging. This may be due to the genomic plasticity of *Candida* species [15,16,17]. Indeed, karyotypic diversity is commonly observed in *Candida* species and isolates [18,19,20,21], and yeast cells can be found as haploids, diploids, and tetraploids, as well as aneuploids [22,23,24] as a consequence of drug-induced genomic instability or as a selective advantage in the presence of the drug [25]. Changes in karyotype patterns and ploidy levels have the potential to cause phenotypic changes that promote adaptation to stress conditions in the environment, host niches or in response to antifungal drugs [15,16]. More recently, matrix-assisted laser desorption ionization time-of-flight mass spectrometry MALDI-TOF MS [26,27] and vibrational spectroscopy, namely FTIR [28,29,30] and Raman [31,32,33] spectroscopy, have been adapted as an accurate, rapid, and inexpensive typing methods for *Candida* species. However, these methods have not yet been comparatively evaluated in a large number of clinical isolates concomitantly with accepted standards and have been used as isolated methods without providing a comparison to karyotype profiling and/or ploidy analysis.

The aim of the present study was to verify the usefulness of FTIR and Raman spectroscopy for *Candida* clinical isolate identification and diagnosis and establish the impact of selected parameters (karyotype polymorphism, DNA content, antifungal treatment) on vibrational spectroscopy-based biochemical profiling. In the present study, 25 clinical *Candida* isolates were characterized for their karyotypic features, DNA content and FTIR spectra- and Raman spectra-based molecular fingerprints and collected data (chromosome number, DNA content, characteristic FTIR and Raman vibrations) were used to create a heat map and group *Candida* isolates. We have documented a joined cluster analysis for proper and successful *Candida* species grouping. The effect of antifungal treatment was also established.

## 2. Results and Discussion

### 2.1. Karyotype Profiling and DNA Content Analysis

**Figure 1:**
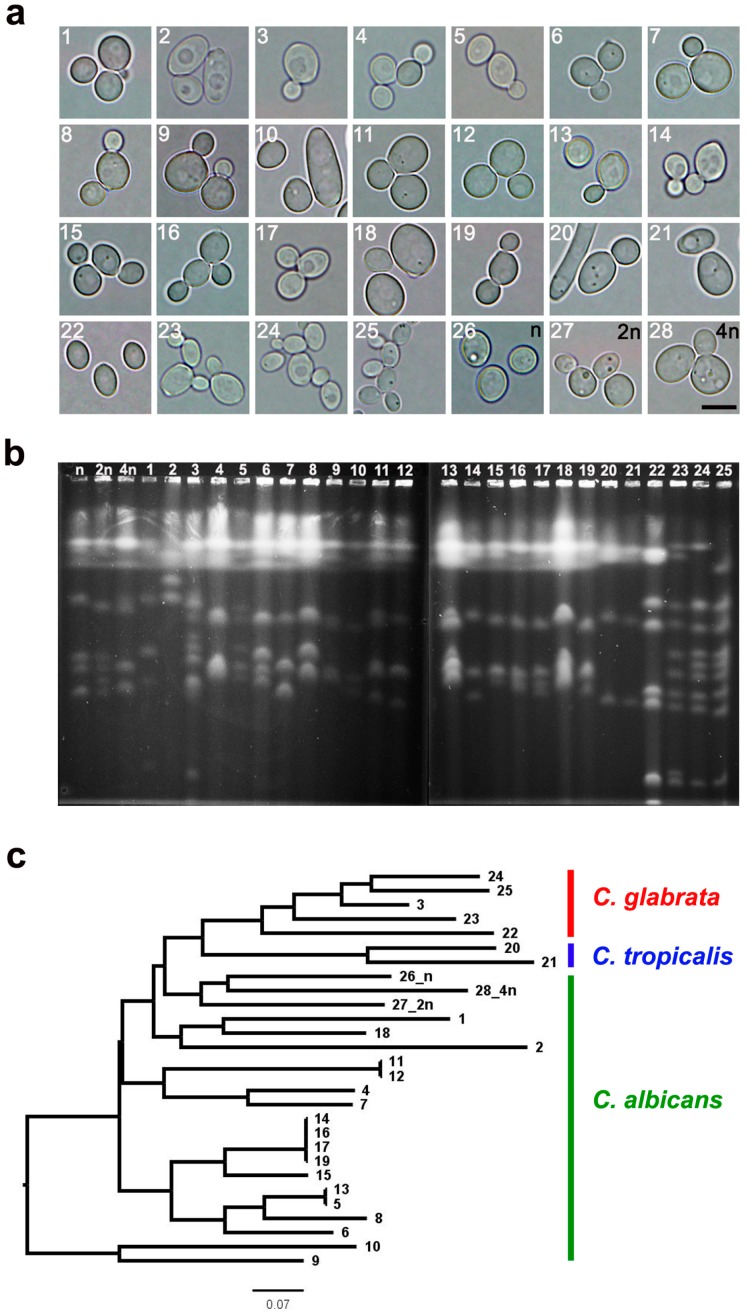
Cell morphology (**a**) and electrophoretic karyotyping (**b**,**c**) of 25 clinical *Candida* isolates and three reference strains (haploid, diploid, and tetraploid). (**a**) Representative microphotographs are shown. Scale bar 5 μm, objective 100×. (**b**) Representative karyotype patterns are shown. (**c**) The dendrogram of chromosome band-based similarity. *C. albicans* group (1, 2, and 4–19), *C. tropicalis* group (20, 21), *C. glabrata* group (3 and 22–25), 302 haploid reference strain (26), SC5314 diploid strain (27), and T15 tetraploid strain (28).

A total of 25 clinical *Candida* samples were classified into three species categories, namely *C. albicans* (samples from 1 to 2 and from 4 to 19, *n* = 18), *C. tropicalis* (samples from 20 to 21, *n* = 2) and *C. glabrata* (sample 3 and samples from 22 to 25, *n* = 5) (Table 1) based on DNA sequencing and a biochemical test. In general, these two assays yielded similar identification results, except of sample 3 that was identified as *C. glabrata* species based on ITS1 sequencing and as *C. albicans* species based on a biochemical test. As DNA sequencing is a more reliable identification method, we assumed that sample 3 is indeed *C. glabrata*. Firstly, morphological features of yeast cells were assessed (Figure 1a).

In general, typical shapes (spherical to oval) and sizes (2–5 × 3–7 µm) of *Candida* cell isolates with the ability to form buds and/or hyphae/pseudohyphae were compared to reference strains (Figure 1a). The ability to grow in yeast, pseudohyphal and hyphal forms is a characteristic feature of *C. albicans* biology [34]. As expected [1,11,35], *C. glabrata* budding cells (blastoconidia) (1–4 µm) were smaller then *C. albicans* (4–6 µm) and *C. tropicalis* cells (4–8 µm) that is due to the fact that *C. glabrata* is generally considered haploid while *C. albicans* and *C. tropicalis* are diploid and *C. glabrata* did not form hyphae/pseudohyphae (Figure 1a).

Of course, clinical species cannot be determined solely based upon morphological features (Figure 1a). CHEF-PFGE was used for karyotype analysis (Figure 1b). *C. albicans* cells have eight pairs of chromosomal homologs, ranging in size from 0.95 to 3.3 Mb and comprising 16 Mb in total [36], but we were able to observe from four to nine chromosomes in clinical isolates assigned to *C. albicans* species (Figure 1b). The reference strains of *C. albicans*, namely haploid (302), diploid (SC5314) and tetraploid (T15) were characterized by seven, eight, and six distinguishable chromosomes, respectively (Figure 1b). This confirms a high genomic diversity of *C. albicans* species [18,36]. It has been suggested that genomic diversity of *C. albicans* is due to chromosome length polymorphism (CLP) that results from expansion and contraction of subrepeats RPS; reciprocal translocation at the major repeat sequence (MRS) loci; chromosomal deletion and trisomy of individual chromosomes [36]. The karyotypes of *C. tropicalis* and *C. glabrata* isolates were found to be much more consistent (Figure 1b). Two *C. tropicalis* isolates had four chromosomes and five *C. glabrata* isolates had from six to twelve distinguishable chromosome bands (Figure 1b). The genome of *C. glabrata* clinical isolates was found to be very plastic with the variations in the number and size of chromosomes and the occurrence of intra- and interchromosomal segmental duplications [37]. For example, it has been reported that *C. glabrata* CBS 138 strain has 13 chromosomes with the genome size of 12.3 Mb [38]. CHEF-PFGE analysis found a minimum of 10 chromosome bands in *C. glabrata* [39]. Moreover, rapid changes in *C. glabrata* genomic organization have been comprehensively documented in numerous clinical studies [40,41,42,43]. Interestingly, isolates from one patient may exhibit 2 or 3 different karyotypes and during infection the chromosome pattern may change within a few days [41].

Phylogenetically, *C. glabrata* is more closely related to the model yeast *Saccharomyces cerevisiae* than to other *Candida* pathogens [38], as *C. glabrata* belongs to post-WGD (whole genome duplication) yeasts [37]. Chromosome similarity between 25 clinical *Candida* isolates was also further evaluated using NJ clustering (this study). Three considered species, namely *C. albicans*, *C*. *tropicalis*, and *C. glabrata* were characterized by clearly separate three clusters (Figure 1c). The most accented chromosome polymorphism was observed among the *C. albicans* group. Nevertheless, due to high genomic diversity and plasticity [15,36,44], it is difficult to discriminate between *Candida* species based on karyotype profiling only.

**Figure 2:**
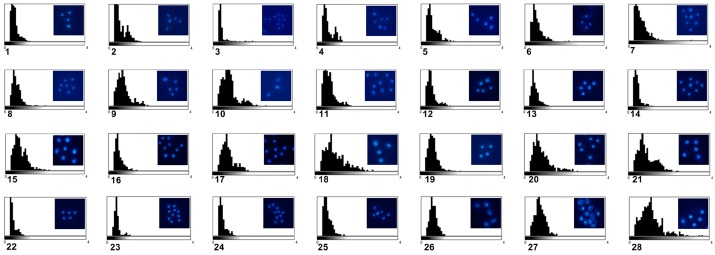
Fluorescence microscopy-based analysis of DNA content. Fixed cells (*n* = 100) were analyzed using an Olympus BX61 fluorescence microscope equipped with a DP72 CCD camera and Olympus CellF software (Olympus, Warsaw, Poland). For DNA visualization, the slides were counterstained with a drop of mounting medium containing 4′,6′-diamino-2-phenylindole (DAPI) (blue). DNA content of clinical *Candida* isolates were compared to reference strains, namely haploid (26), diploid (27), and tetraploid (28) strains. DNA content was expressed as arbitrary units (relative fluorescence units from 0 to 4). Representative microphotographs and data distribution (histograms) are shown. *C. albicans* group (1, 2, and 4–19), *C. tropicalis* group (20, 21), *C. glabrata* group (3 and 22–25), 302 haploid reference strain (26), SC5314 diploid strain (27), and T15 tetraploid strain (28).

We also analyzed DNA content of *Candida* isolates using fluorescent measurements and compared them to the reference strains used (n, 2n, and 4n) (Figure 2). However, one should remember that haploid strains of *C. albicans* are considered to be unstable, often autodiploidize, and that genomic features often vary among tetraploid strains as well [23,45].

As expected [46], DNA content of *C. glabrata* isolates was found to be the lowest (Figure 2). DNA content of *C. tropicalis* isolates was higher than that of *C. albicans* and DNA content of *C. albicans* isolates was enormously diverse with a broad range between minimal and maximal values (Figure 2). When considered relative fluorescent units (arbitrary units), n, 2n, and 4n reference strains were characterized by mean arbitrary units of 0.6, 0.74, and 1.07, respectively, whereas *C. glabrata*, *C. tropicalis*, and *C. albicans* isolates were characterized by mean arbitrary units of 0.46, 0.87, and 0.62, respectively (Figure 2). Our data confirm DNA content diversity and plasticity of *Candida albicans* [16,44]. The DNA content/ploidy variation is considered as an adaptive mechanism in human pathogenic fungi [16,44,47]. *C. albicans* is normally a diploid organism (2n = 16), but a variety of stresses, namely heat shock, antifungal drug treatment or host-pathogen interactions can stimulate a plethora of aneuploidy events that seems to be well tolerated and may be considered as a selectively advantageous, e.g., may promote antifungal drug resistance [16,44,47]. Indeed, a specific segmental aneuploidy, consisting of an isochromosome composed of the two left arms of chromosome 5 (i5L), was reported to be associated with azole resistance in *C. albicans* [48]. This was achieved by amplification of two genes involved in fluconazole resistance, namely *ERG11* (that encodes lanosterol–14-α-demethylase, the target of fluconazole) and *TAC1* (that encodes a transcriptional regulator of ABC-transporter drug efflux pumps Cdr1 and Cdr2 that reduce intracellular azole concentration) [49]. More recently, trisomy of chromosome R and trisomy of chromosome 4 have been also reported to contribute to azole resistance in *C. albicans* [50,51]. The genomic plasticity is also associated with antifungal drug resistance in *C. glabrata* as the formation of new chromosomes was established as a virulence mechanism in *C. glabrata* clinical isolates [43]. Surprisingly, spontaneous changes in ploidy are also widespread in nonpathogenic fungi, namely in the model yeast *Saccharomyces cerevisiae* [52]. The appearance of diploid cells among haploid yeast cultures evolving for over 100 generations was documented and spontaneous diploidization was observed [52]. This relatively common event was based on both whole genome duplication (endoreduplication) and mating-type switching despite the use of heterothallic strains [52]. It has been suggested that spontaneous diploidization can be advantageous under certain stressful conditions in budding yeast [52]. Chromosomal copy number changes were also observed while analyzing the genome of clinical *Saccharomyces cerevisiae* strains that highlights the potential importance of large-scale genomic copy variation in yeast adaptation [53].

### 2.2. Biochemical Features Reflect Genomic Diversity and Plasticity of Candida Cells

**Figure 3:**
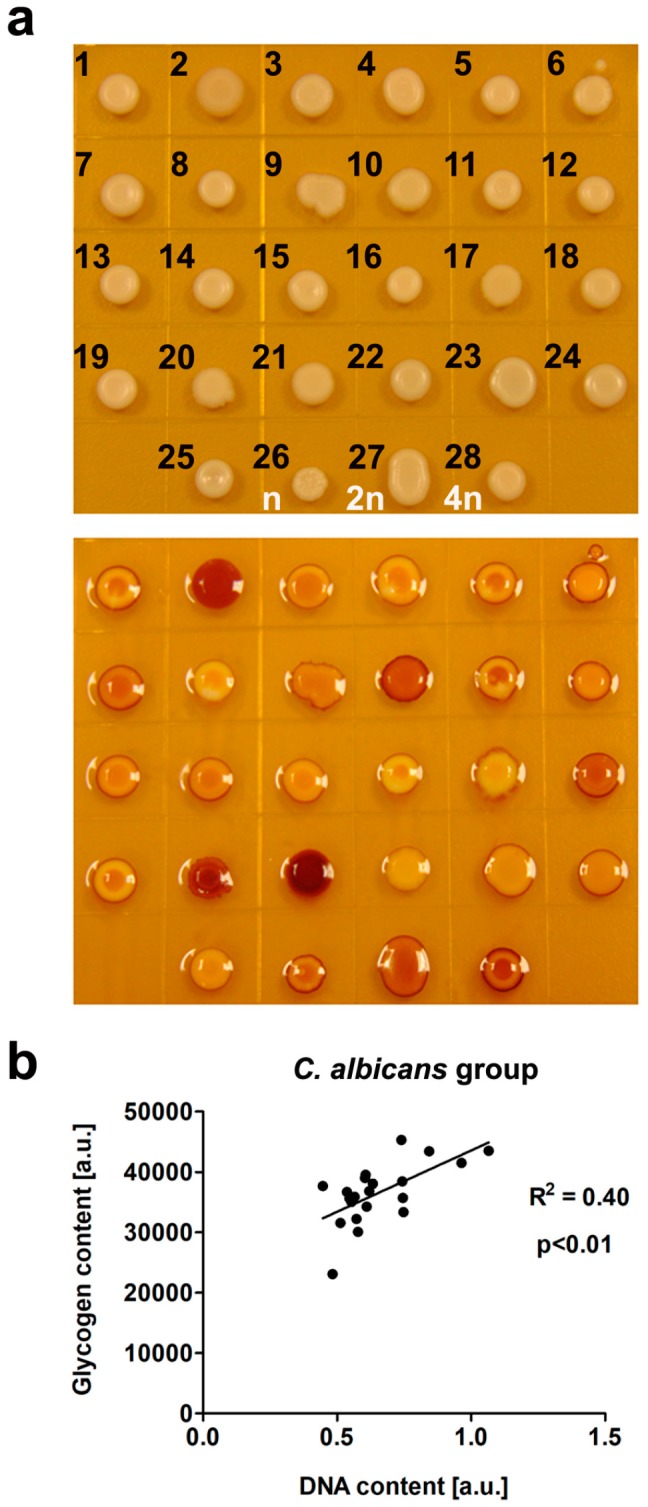
Examination of glycogen content by iodine staining (**a**, lower panel). Unstained *Candida* cells (spots) are also presented for comparison (**a**, upper panel). *C. albicans* group (1, 2, and 4–19), *C. tropicalis* group (20, 21), *C. glabrata* group (3 and 22–25), 302 haploid reference strain (26), SC5314 diploid strain (27), and T15 tetraploid strain (28). (**b**) Correlation analysis between glycogen content [a.u.] and DNA content [a.u.] within *C. albicans* group is shown. Correlation analysis of the data was performed using a linear correlation (Pearson r) test. The R^2^ value is shown.

We have then analyzed some biochemical features of clinical *Candida* isolates and we focused on elucidation of the usefulness of FTIR and Raman spectroscopy for *Candida* clinical isolate identification and determination of the effects of selected traits, such as karyotype polymorphism and DNA content on vibrational spectroscopy-based biochemical profiling. Initially, we considered the ability of *Candida* cells to accumulate glycogen (Figure 3).

**Figure 4:**
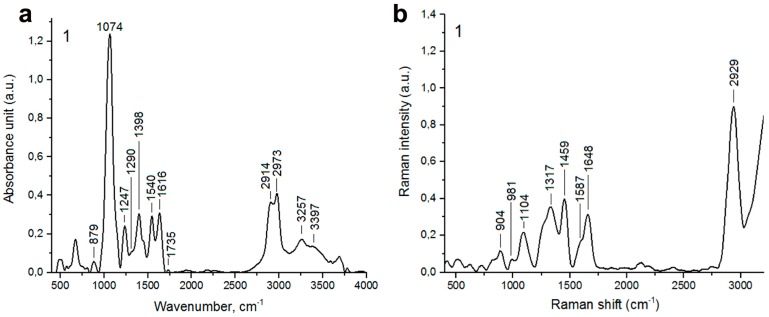
A representative FTIR spectrum (**a**) and Raman spectrum (**b**) of a clinical *Candida albicans* isolate 1. Characteristic peaks are denoted; a.u., arbitrary units. A comma was used as a decimal separator.

We found that higher DNA content was correlated with higher glycogen storage as judged using *C. albicans* reference strains of different ploidy, namely n, 2n, and 4n cells (Figure 3b). Moreover, isolates of *C. glabrata* with relatively low DNA content were characterized by the lowest ability to accumulate glycogen (Figure 3a). In contrast, clinical *C. tropicalis* isolates with higher DNA content compared to *C. glabrata* cells (Figure 2) were found to accumulate the highest levels of glycogen among *Candida* cells considered (Figure 3a). Additionally, *C. albicans* samples, namely isolates 2, 10, and 18, with much higher DNA content compared to other *C. albicans* samples were characterized by much higher ability to accumulate glycogen (Figure 3a). As the DNA content may reflect biochemical/metabolic features in *Candida* isolates, we have then considered more sophisticated biochemical profiling using both FTIR and Raman spectroscopy (Figure 4).

In general, FTIR spectroscopy and Raman spectroscopy are used for analytical chemistry applications. More recently, vibrational spectroscopy has been used to characterize biological materials, especially in the field of biomedicine for the rapid differentiation, classification, identification and large-scale screening at subspecies level of clinically relevant microorganisms [54,55,56,57]. These reagentless and nondestructive techniques are based on the absorption (FTIR) or scattering (Raman) of light directed onto a sample and provide a highly specific spectroscopic fingerprints of microorganisms by which they can be identified [54,55,56,57], and also enable for a detailed structural analysis to identify certain intracellular macromolecules [58]. However, data on vibrational spectroscopic identification of clinical *Candida* isolates in parallel with karyotype profiling, DNA content analysis and routine diagnostic phenotypic identification are not available.

**Figure 5:**
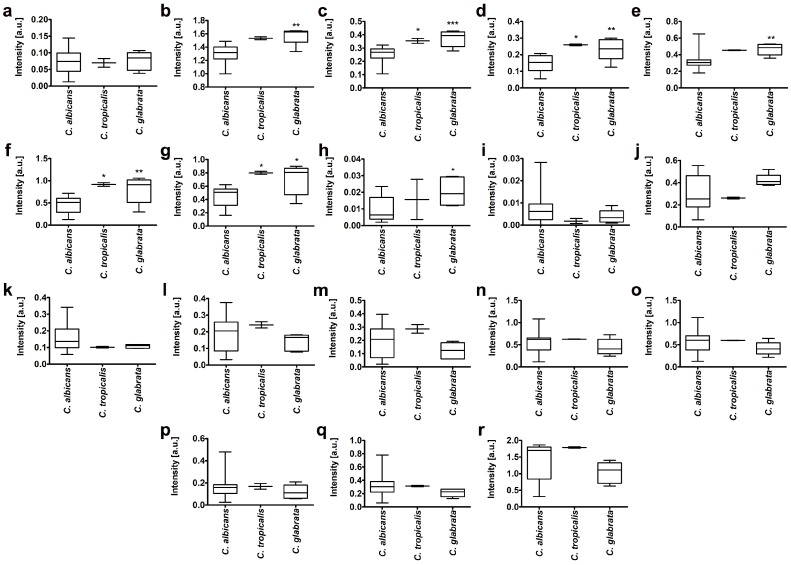
A comparative analysis of differences in the intensities of some FTIR (**a**–**k**) and Raman (**l**–**r**) vibrations between *C. albicans* group (1, 2, and 4–19), *C. tropicalis* group (20, 21), *C. glabrata* group (3 and 22–25), reference strain group (haploid, 26; diploid, 27; and tetraploid, 28); a.u., arbitrary units. (**a**) C-O, C-O-H, and C-O-C deformation and C–C stretching vibrations from carbohydrates, (**b**) β(1–3) glucans, nucleic acids and glycogen, PO^2−^ symmetric stretching vibrations mainly from RNA, (**c**) C–O asymmetric stretching vibrations from phospholipids and lipids, (**d**) amide III: C–N and C–O stretching vibrations, N–H and O=C–N bending vibrations, (**e**) C=O of COO^−^ symmetric stretching vibrations from proteins, CH_2_ wagging vibrations from lipids and β(1–3) glucans, (**f**) amide II: mainly C–N stretching vibrations and N–H bending vibrations, (**g**) amide I: mainly C=O stretching vibrations and contributions of N–H bending vibrations, (**h**) C=O stretching vibrations from lipid esters, (**i**) CH_2_ stretching vibrations from lipids, (**j**) CH_3_ stretching vibrations from lipids, (**k**) amide A from proteins, (**l**) symmetric benzene/pyrrole in-phase and out of phase breathing mode of tryptophan and phenylalanine, (**m**) symmetric benzene/pyrrole in-phase and out of phase breathing mode of tryptophan and phenylalanine, (**n**) C–H deformation vibrations from proteins, (**o**) C–H deformation vibrations from lipids, (**p**) ring stretching vibrations from the deoxyribonucleotide adenosine monophosphate, (**q**) Amide I: mainly C=O stretching vibrations and contributions of N–H bending vibrations, (**r**) C-H stretching vibration from lipids. Box and whisker plots are shown, *** *p* < 0.001, ** *p* < 0.01, * *p* < 0.05 compared to *C. albicans* group (ANOVA and Tukey’s a posteriori test).

Typical FTIR and Raman spectra of *Candida* isolate 1 with marked individual vibrations corresponding to functional groups of nucleic acids, phospholipids, carbohydrates, proteins, and lipids are presented in Figure 4. In the FTIR spectrum (Figure 4a), peaks at wavenumbers 879 cm^−1^ and 1074 cm^−1^ are corresponding to C-O, C-O-H, and C-O-C deformation and C-C stretching vibrations of carbohydrates and β(1–3)glucans, nucleic acids and glycogen and PO^2−^ symmetric stretching vibrations mainly from RNA [58,59,60], respectively. The peak at 1247 cm^–1^ originates from C-O asymmetric stretching vibrations in phospholipids [59]. Moreover, the vibration at 1290 cm^−1^ corresponds to amide III [59]. In the FTIR spectrum, peak at 1398 cm^−1^ originates from C=O of COO^−^ symmetric stretching vibrations in proteins and CH_2_ wagging vibrations in lipids and β(1–3)glucans [59]. The peaks at 1540 cm^−1^ and 1616 cm^−1^ correspond to amide II and amide I vibrations, respectively [58]. Furthermore, peaks at: 1735 cm^−1^, 2914 cm^−1^, and 2973 cm^−1^ originate from CH vibrations in lipids [61]. The last two peaks in the FTIR spectrum (3257 cm^−1^ and 3397 cm^−1^) correspond to OH vibrations from water and amide A from proteins, respectively [62]. Moreover, in the Raman spectrum (Figure 4b), the vibrations from symmetric benzene/pyrrole in-phase and out-of-phase breathing modes of tryptophan and phenylalanine (904 cm^−1^, 981 cm^−1^) are observed. Furthermore, peaks at 1317 cm^−1^ and 1459 cm^−1^ correspond to C-H deformation vibrations from proteins [63] and C-H deformation vibrations from lipids [63], respectively. In the Raman spectrum, a vibration at 1587 cm^−1^ originating from ring stretching vibrations of the deoxyribonucleotide adenosine monophosphate is observed [63]. Amide I vibrations in Raman spectrum are documented at 1648 cm^−1^ [63]. Moreover, a peak at 2929 cm^−1^ originates from C-H stretching vibration from lipids is observed [64]. FTIR and Raman spectra of all clinical isolates considered are presented in Supplemental Figure S1 and Supplemental Figure S2. The peak positions and information about vibrations for all samples are denoted in Supplemental Table S1. According to FTIR and Raman spectra, the differences in the signal intensity of functional groups as well as differences in the occurrence of these groups may be noticed (Supplemental Figure S1 and Supplemental Figure S2 and Supplemental Table S1). According to the differences in signal intensities of some selected vibrations of FTIR and Raman spectra, we have performed a comparative analysis between clinical samples belonging to three *Candida* species (Figure 5). We have considered 11 vibrations of the FTIR spectrum and seven vibrations of the Raman spectrum (Figure 5).

For seven vibrations of FTIR spectrum, we were able to obtain statistically significant differences in signal intensities between *C. albicans* isolates and other *Candida* isolates. These vibrations were: vibrations from β(1–3) glucans, nucleic acids and glycogen, PO^2−^ symmetric stretching vibrations mainly from RNA; C-O asymmetric stretching vibrations from phospholipids and lipids; amide III: C–N and C–O stretching vibrations, N–H and O=C–N bending vibrations; C=O of COO^−^ symmetric stretching vibrations from proteins, CH_2_ wagging vibrations from lipids and β(1–3)glucans; amide II: mainly C–N stretching vibrations and N–H bending vibrations, amide I: mainly C=O stretching vibrations and contributions of N–H bending vibrations; C=O stretching vibrations from lipid esters (Figure 5). Protein components of clinical *Candida* isolates were also characterized. To determine a secondary protein structure, a deconvolution of FTIR amide I region was considered (Supplemental Figure S3). The abundance of α and β structures, as well as the ratio of α/β structures within analyzed peak were calculated (Supplemental Table S2). *C. tropicalis* group was characterized by higher ratio of α/β structures compared to other *Candida* groups (Supplemental Table S2). The lipid-carbohydrate ratio was also analyzed in clinical *Candida* isolates (Supplemental Table S3) that was calculated on the basis of peak area corresponding to lipid and carbohydrate vibrations (Supplementary Table S4). In general, within *C. tropicalis* and *C. glabrata* groups, the lipid-carbohydrate ratio was comparable and the variability was rather slightly accented. In contrast, *C. albicans* group was characterized by a diverse lipid-carbohydrate ratio, e.g., ranging from 0.16 to 0.93 (Supplemental Table S3).

**Figure 6:**
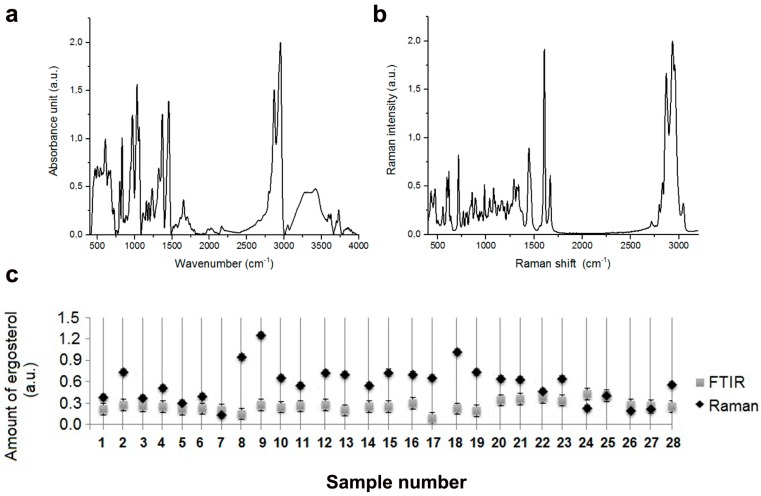
FTIR spectrum (**a**) and Raman spectrum (**b**) of ergosterol reference standard. (**c**) Quantitative analysis of ergosterol content in clinical *Candida* isolates based on both FTIR and Raman spectroscopy. *C. albicans* group (1, 2, and 4–19), *C. tropicalis* group (20, 21), *C. glabrata* group (3 and 22–25), 302 haploid reference strain (26), SC5314 diploid strain (27), and T15 tetraploid strain (28). Bars indicate SD, *n* = 3, a.u., arbitrary units.

More recently, the quantitation of ergosterol content has been established as a novel method for determination of fluconazole susceptibility of *C. albicans* [65]. Azole stress has been also reported to cause upregulation of genes involved in sterol uptake and biosynthesis in *C. glabrata* [66]. Fluconazole treatment resulted in increased mRNA levels of ergosterol biosynthetic genes, namely *CgERG2*, *CgERG3*, *CgERG4*, *CgERG10*, and *CgERG11* and sterol influx transporter *AUS1* and sterol metabolism regulators *SUT1* and *UPC2* in *C. glabrata* [66]. Moreover, stimulation with exogenous source of cholesterol or ergosterol conferred resistance to fluconazole and voriconazole in *C*. *glabrata* [66]. As ergosterol abundance may modulate azole antifungal resistance in clinical *Candida* isolates, we decided then to analyze ergosterol content using both FTIR and Raman spectroscopy (Figure 6).

Representative FTIR and Raman spectra of ergosterol are presented in Figure 6a,b, respectively. Using FTIR spectroscopy, similar levels of ergosterol were revealed in all analyzed samples (Figure 6c). However, using Raman spectroscopy, we were able to show differences in the content of ergosterol (Figure 6c). For ergosterol content analysis, we have selected a peak at 1459 cm^−1^ instead of a peak at 1602 cm^−1^ [67] to rule out the possibility of some overlapping with protein signals. The most diverse group in term of ergosterol content was *C. albicans* group, e.g., sample 7 was characterized by eight times lower levels of ergosterol than sample 9 (Figure 6c).

**Figure 7:**
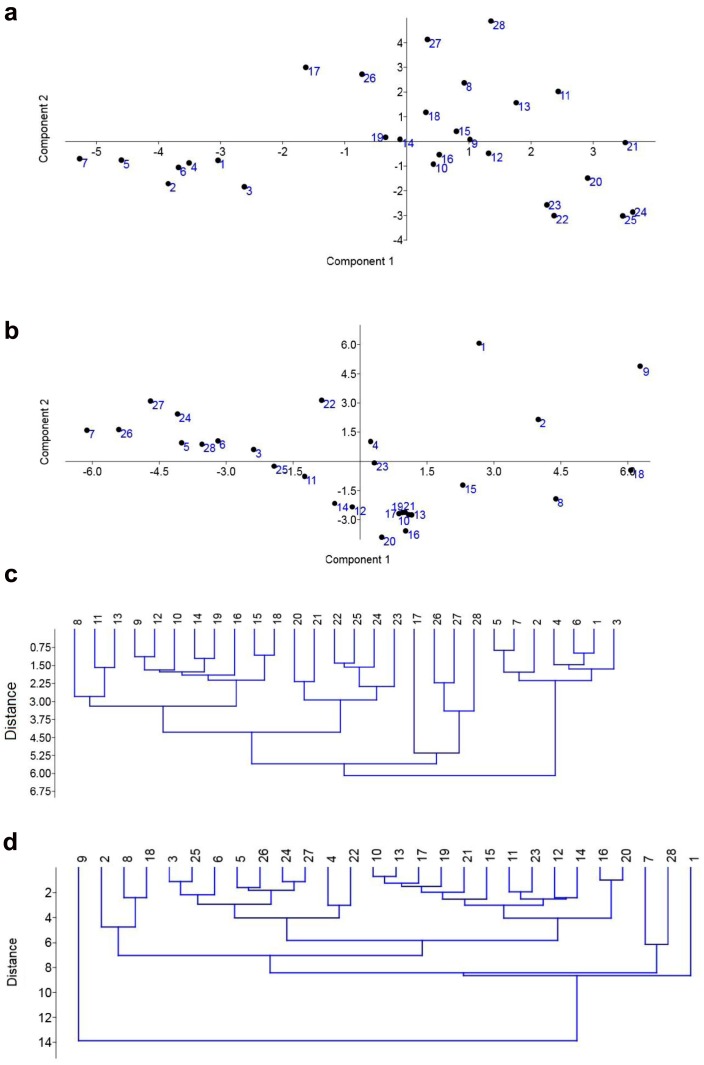
PCA two-dimensional score plot of FTIR spectra (**a**) and Raman spectra (**b**) for principle components (PA) 1 and 2 based on lipid-carbohydrate ratio and α/β structure ratio. HCA analysis based on FTIR spectra ranging from 500 to 4000 cm^−1^ (**c**) and Raman spectra ranging from 500 to 3000 cm^−1^ (**d**). Representative dendrograms are shown. *C. albicans* group (1, 2, and 4–19), *C. tropicalis* group (20, 21), *C. glabrata* group (3 and 22–25), 302 haploid reference strain (26), SC5314 diploid strain (27), and T15 tetraploid strain (28).

We have then considered principal component analysis (PCA) and hierarchical cluster analysis (HCA) using both FTIR and Raman spectra (Figure 7).

For PCA, we have selected lipid-carbohydrate ratio and α/β structure ratio (Figure 7a,b). According to FTIR spectra (Figure 7a), isolates from *C. tropicalis* (samples 20 and 21) and *C. glabrata* (samples 22 to 25, but not sample 3) species were grouped together within their own categories, whereas *C. albicans* group was found to be diverse with several separated subgroups, e.g., a subgroup that consists of samples 1, 2, 4–7 or a subgroup that consists of samples 14, 17, and 19 that was also grouped with a haploid reference strain (sample 26). Raman spectra-based PCA did not reveal similar clustering (Figure 7b). We have then considered HCA based on FTIR spectra from 500 to 4000 cm^−1^ and Raman spectra from 500 to 3000 cm^−1^ (Figure 7c,d). According to FTIR spectra, *Candida* isolates were grouped into previously assigned species, namely *C. albicans*, *C. tropicalis*, and *C. glabrata* (Table 1, Figure 7c). Similarly to PCA, several subgroups of *C. albicans* group were documented, e.g., one containing samples from 1 to 2 and from 4 to 7 and second with samples from 8 to 19 without sample 17 that was grouped as its own category (Figure 7c). In general, such clustering also reflected the differences in DNA content among *C. albicans* group (Figure 2). In contrast, Raman spectra-based HCA did not provide discrimination between *Candida* species (Figure 7d).

There are several reports on vibrational spectroscopy identification of clinical *Candida* isolates, but none of them provide a comparison with karyotype profiling and DNA content analysis. The difficulty of differentiating at the strain level, especially when high accumulated doses of an antifungal agent are involved, has been documented while analyzing FTIR spectra of pathogenic *C. albicans* isolates from HIV-positive patients [28]. Using FTIR spectroscopy, six species (*C. albicans*, *C. glabrata*, *C. parapsilosis*, *C. tropicalis*, *C. krusei*, and *C. kefyr*) from a collection of 57 clinical strains of *Candida* and isolated from hospitalized patients were identified with a classification rate of 100% for both microcolonies and 24 h cultures [29]. More recently, next generation sequencing (NGS) of ITS and D1/D2 LSU marker regions together with FTIR spectroscopy were applied to identify 256 pathogenic strains belonging to *Candida* genus [30]. Strains of *C. albicans*, *C. parapsilosis*, *C*. *glabrata*, and *C. tropicalis* were identified with high-throughput NGS sequencing of ITS and LSU markers and then with FTIR, and total percentage of correct identification reached 97.4% for *C. albicans* and 74% for *C. parapsilosis* while the other two species showed lower identification rates [30]. The authors concluded that the identification success increases with the increasing number of strains actually used in the PLS analysis [30].

A set of 42 *Candida* strains comprising five species that are frequently encountered in clinical microbiology was also considered to analyze the usefulness of confocal Raman microspectroscopy for the rapid identification of *Candida* species [32]. Using multivariate statistical analyses, a high prediction accuracy (97 to 100%) was documented [32]. The authors concluded that confocal Raman microspectroscopy offers a rapid, accurate, and easy-to-use alternative for the identification of clinically relevant *Candida* species [32]. Raman spectroscopy has been also found an accurate and rapid (12–24 h) alternative for the identification of *Candida spp*. in peritonitis patients [31].

**Figure 8:**
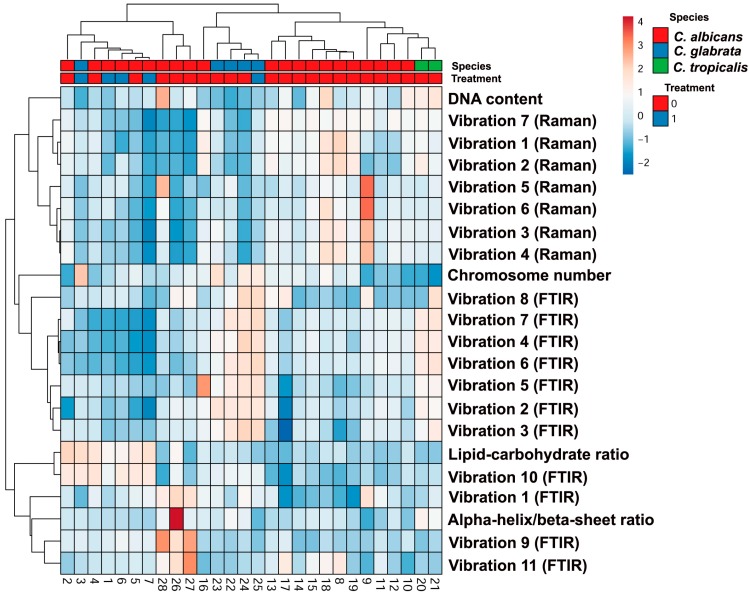
A joined clustering analysis of chromosome number, DNA content, signal intensities of some selected vibrations of FTIR and Raman spectra (11 vibrations of FTIR spectrum, seven vibrations of Raman spectrum), alpha-helix/beta-sheet ratio and lipid-carbohydrate ratio that allows for proper grouping of three *Candida* species considered. The effect of antifungal treatment is also denoted. A heat map generated from FTIR and Raman spectroscopy data, karyotype profiling, and DNA content data is shown. Hierarchical clustering was created using ClustVis, a web tool for visualizing clustering of multivariate data (BETA) (https://biit.cs.ut.ee/clustvis/). FTIR vibrations: (1) C–O, C–O–H, and C–O–C deformation and C–C stretching vibrations from carbohydrates, (2) β(1–3) glucans, nucleic acids and glycogen, PO^2–^ symmetric stretching vibrations mainly from RNA, (3) C–O asymmetric stretching vibrations from phospholipids and lipids, (4) amide III: C–N and C–O stretching vibrations, N–H and O=C–N bending vibrations, (5) C=O of COO^−^ symmetric stretching vibrations from proteins, CH_2_ wagging vibrations from lipids and β(1–3)glucans, (6) amide II: mainly C–N stretching vibrations and N–H bending vibrations, (7) amide I: mainly C=O stretching vibrations and contributions of N–H bending vibrations, (8) C=O stretching vibrations from lipid esters, (9) CH_2_ stretching vibrations from lipids, (10) CH_3_ stretching vibrations from lipids, (11) Amide A from proteins; Raman vibrations: (1) symmetric benzene/pyrrole in-phase and out of phase breathing mode of tryptophan and phenylalanine, (2) symmetric benzene/pyrrole in-phase and out of phase breathing mode of tryptophan and phenylalanine, (3) C–H deformation vibrations from proteins, (4) C–H deformation vibrations from lipids, (5) ring stretching vibrations from the deoxyribonucleotide adenosine monophosphate, (6) amide I: mainly C=O stretching vibrations and contributions of N–H bending vibrations, (7) C–H stretching vibration from lipids.

Finally, we have considered a joined clustering analysis of chromosome number, DNA content, the intensities of 11 vibrations of FTIR spectrum and seven vibrations of Raman spectrum, alpha-helix/beta-sheet ratio, and lipid-carbohydrate ratio (Figure 8).

Using this approach, we were able to discriminate between *Candida* species considered (Figure 8). One exception was sample 3, *C. glabrata* species that was classified within *C. albicans* group (Figure 8). However, this result may be due to fluconazole treatment (Table 1, Figure 8).

**Figure 9:**
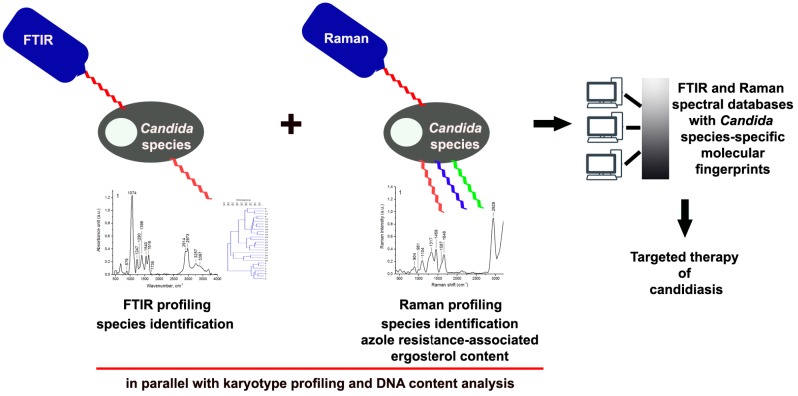
The usefulness of vibrational spectroscopy methods for comprehensive biochemical characterization and identification of clinical *Candida* isolates. FTIR spectra- and Raman spectra-based biochemical profiling of clinical *Candida* isolates together with karyotype profiling and DNA content analysis allows for accurate identification of *Candida* species. Raman spectroscopy can be also adapted for rapid and accurate measurements of ergosterol content that may provide information of azole resistance/susceptibility. Established spectral databases can be useful for diagnosis and targeted therapy of candidiasis.

Taken together, we have shown for the first time that vibrational spectroscopy-based biochemical profiling reflected genomic diversity (karyotype patterns, DNA content) of 25 clinical *Candida* isolates (Figure 8). However, using FTIR or Raman spectroscopy as isolated methods for *Candida* species identification may be limited. FTIR- as well as Raman-based clustering analysis (Figure 7) yielded ambiguous results that were not entirely comparable to karyotype profiling-based clustering analysis (Figure 1c). Thus, only joined clustering analysis of chromosome number, DNA content and vibrational spectroscopy-based biochemical profiling may allow for grouping together the clinical *Candida* isolates from the same species (Figure 8). The usefulness of vibrational spectroscopy methods for characterization and identification of clinical *Candida* isolates is also summarized in Figure 9.

We also postulate that Raman spectroscopy can be adapted for rapid and accurate analysis of ergosterol content in clinical *Candida* isolates and thus may provide information on azole resistance/susceptibility (Figure 9). Vibrational spectroscopy-based data may be included in global spectral databases for identification purposes and may facilitate diagnosis and targeted therapy of candidiasis (Figure 9). Indeed, limited use of vibrational spectroscopy-based techniques for medical diagnostics seems to be due to the absence of reliable and validated libraries linked to taxonomically sound identification procedure [30]. More recently, it has been postulated that such libraries should include several tens of strains for each relevant species and the panel of strains needs to be composed of well-identified strains, e.g., deriving from diverse sources and collected over an extensive time period [30]. Postulated approach would require a multidisciplinary effort of specialists working in strain isolation and maintenance, molecular taxonomy, vibrational spectroscopy-based techniques, data management and data basing [30].

## 3. Materials and Methods

### 3.1. Ethics Statement

This study was approved by the Ethics Committee of the Faculty of Medicine, University of Rzeszow, Poland (approval code 2018/06/03, approved on 14 June 2018). All samples were analyzed anonymously. Clinical *Candida* species isolates were obtained from the Clinical Microbiology Laboratory (Department of Diagnostic Medicine, Provincial Medical Specialist Unit, Rzeszow, Poland).

### 3.2. Clinical Specimens and Reference Strains

A total of 25 clinical samples used in this study are listed in Table 1. Clinical isolates were originated from human bronchoalveolar lavage, sputum, pharynx, wound, urine, and vagina. Patient 1 and patients 3, 6, 8, and 25 had been treated with voriconazole and fluconazole, respectively (Table 1). The following three *Candida albicans* strains of known ploidy were used as reference strains: *C. albicans* 302 (haploid), *C. albicans* SC5314 (diploid), and *C. albicans* T15 (FH6, tetraploid but trisomic for chromosomes 2/3 with multiple copies of chromosome 5L) [49]. The reference strains were a generous gift from Prof. Judith Berman (Department of Molecular Microbiology and Biotechnology, Tel Aviv University, Israel).

### 3.3. Culture Conditions and Species Identification

For identification of *Candida* species, clinical samples were diluted in 0.9% NaCl (Sigma-Aldrich, Poznan, Poland) and evaluated under a light microscope, e.g., the presence of hyphae, pseudohyphae and yeast cells was considered, and spread onto Sabouraud Dextrose Agar (SDA) plates (bioMerieux, Warsaw, Poland). Yeast cells were cultured at 25 °C and at 37 °C for seven days and morphological features were then assessed. Species identification was based on the API^®^ *Candida* biochemical test (bioMerieux, Warsaw, Poland) and DNA sequencing. For DNA isolation, clinical isolates were inoculated in 20 mL of Sabouraud dextrose broth (BTL, Lodz, Poland) under shaking conditions at 29 °C to obtain log-phase cultures. Five milliliters of overnight culture was centrifuged (5000× *g*) at 4 °C for 10 min. The cells were washed twice with 20 mM phosphate buffer, 1 mM EDTA, pH 7.5 and resuspended in 1 mL of extraction buffer (1 M sorbitol, 0.1 M EDTA, pH 7.5). For spheroplast preparation, zymolyase (1 mg/mL) was then added and samples were incubated at 37 °C for 30 min. The spheroplasts were then centrifuged and resuspended in 500 μL of lysis buffer (50 mM Tris-HCl pH 7.5, 20 mM EDTA, pH 8.0) containing 50 μL of 10% SDS. The mixture was incubated at 65 °C for 10 min and then 200 μL of 5 M potassium acetate was added. The samples were incubated on ice for 5 min and centrifuged (13,000 rpm) for 10 min. The supernatant was transferred to a new tube and then an equal volume of isopropanol was added. Samples were centrifuged (13,000 rpm) for 10 min and the supernatant was subsequently removed. DNA pellets were washed using 95% and 70% ethanol and then dried in a SpeedVac (Thermo Fisher Scientific, Warsaw, Poland). Obtained DNA samples were resuspended in 40 μL of ultrapure DNase⁄RNase-free distilled water and stored at −20 °C until use. The genomic DNA concentration and purity were assessed by using A260/A280 ratio using NanoDrop™ 2000 Spectrophotometer (Thermo Fisher Scientific, Warsaw, Poland). ITS-PCR was carried out in 50 μL reaction volume using ready to use PCR Master Mix (A&A Biotechnology, Gdynia, Poland) and ITS1 primer (5′-TCCGTAGGTGAACCTGCGG-3′, Genomed, Warsaw, Poland) and ITS4 primer (5′-TCCTCCGCTTATTGATATGC-3′, Genomed, Warsaw, Poland). Each reaction mixture contained 300 ng of DNA, 1x PCR Mix with 0.5 mM dNTP (dATP, dCTP, dGTP, dTTP), 2.5 U recombinant DNA polymerase, and 0.1 μM of forward (ITS1) and reverse (ITS4) primers. Eppendorf Mastercycler^TM^ PCR system (Eppendorf, Warsaw, Poland) was used with initial denaturation at 95 °C for 3 min, followed by 30 cycles of denaturation at 95 °C for 30 s, annealing at 55°C for 1 min, extension at 72 °C for 1 min and a final extension step at 72 °C for 5 min. The products of Sanger sequencing were subjected to capillary electrophoresis (Genomed, Warsaw, Poland) and the sequencing reads were analyzed using NCBI nucleotide sequence database (BLASTN option).

Drug susceptibility was evaluated using an ATB FUNGUS 3 strip (bioMerieux, Warsaw, Poland) that consists of 16 pairs of cupules including two growth control wells and five antifungal drugs at different concentrations: 5-flucytosine (4–16 µg/mL), amphotericin B (0.5–16 µg/mL), fluconazole (1–128 µg/mL), itraconazole (0.125–4 µg/mL), and voriconazole (0.06–8 µg/mL). After identification, *C. albicans*, *C. tropicalis*, and *C. glabrata* cells were routinely cultured using YPD medium (1% *w*/*v* Difco Yeast Extract, 2% *w*/*v* Difco Yeast Bacto-Peptone, 2% *w*/*v* dextrose) (BD Biosciences, Sparks, MD, USA) from single colonies either on liquid YPD medium or on solid YPD medium containing 2% *w*/*v* Difco Bacto-agar, at 28 °C. For vibrational spectroscopy-based biochemical profiling, 4 μL of *Candida* cell suspensions at 10^8^ cells/mL were used.

### 3.4. Preparation of Agarose-Embedded Yeast DNA

Yeast DNA, isolated from cells at a logarithmic phase of growth (3 × 10^8^ cells), was obtained using BIORAD CHEF Yeast Genomic DNA Plug Kit (BIORAD, Warsaw, Poland) using a standard protocol [15] according to the manufacturer’s instructions, with minor modifications. Briefly, instead of standard lyticase solution, a mix of standard lyticase and zymolyase 100T, 125 μg/mL (US Biological, Salem, MA, USA), and overnight incubation at 37 °C was used for spheroplast preparation and prolonged proteinase K treatment (48 h at 50 °C) was applied for protein digestion.

### 3.5. Pulsed-Field Gel Electrophoresis (PFGE)

Contour clamped homogeneous electric field (CHEF)-PFGE separation of yeast whole chromosomes was performed on a 1% agarose gel in 0.5× TBE according to the manufacturer’s instructions using CHEF-DR^®^III Pulsed Field Electrophoresis System (BIORAD, Warsaw, Poland) and the following conditions: 60 to 120 s switch, 6 V cm^−1^, 120° angle for 36 h, followed by 120 to 300 s switch, 4.5 V cm^–1^, 120° angle for 12 h. After CHEF-PFGE separation, yeast chromosomes were stained using ethidium bromide. The dendrogram of chromosomal DNA-based similarity was created using Free-Tree software [68] using neighbor-joining (NJ) method with Sokal-Sneath-Anderberg matrix and FigTree tree figure drawing tool (http://tree.bio.ed.ac.uk/software/figtree/) (access on 12 September 2018).

### 3.6. DNA Content Analysis

Yeast cells from log phase cultures were diluted to 10^7^ cells/mL and fixed with 70% ethanol at −21 °C for 24 h. After incubation, the cells were washed with PBS and resuspended in 500 μL of spheroplast buffer, spread onto slides and permeabilized with PBS containing 0.1% Triton X–100. The slides were treated with 100 μg/mL RNAse (Sigma-Aldrich) in 2× saline sodium citrate (SSC) buffer in a humidified chamber at 37 °C for 1 h for enhanced results. Next, the slides were washed three times in PBS buffer. For DNA visualization, the slides were counterstained with a drop of mounting medium containing 4′,6′-diamino-2-phenylindole (DAPI) (Cambio, Cambridge, UK) and then analyzed using an Olympus BX61 fluorescence microscope equipped with a DP72 CCD camera and Olympus CellF software (Olympus, Warsaw, Poland). The CCD capture conditions were as the following: exposure time 150 ms, 100× oil immersion objective. DAPI fluorescent signals were collected using DAPI filters (λ_ex_ = 345 nm, λ_em_ = 455). Fluorescence microscopy was adapted for DNA content analysis. ImageJ software (http://rsbweb.nih.gov/ij/) (access on 29 July 2018). was used to analyze the nuclear DNA content. DNA content was expressed as arbitrary units [a.u.].

### 3.7. Glycogen Storage Assay

The ability of yeast cells to accumulate glycogen was evaluated using iodine staining of yeast colonies [69] on the basis that glycogen gives a reddish-brown coloration with iodine. Briefly, 2 µL of *Candida* cell suspensions at 10^7^ cells/mL were inoculated on solid YPD medium and glycogen storage was detected by flooding 3-day colonies with 5 mL of iodine solution (0.2% I_2_ in 0.4% KI). The staining reactions of the colonies were recorded 1 min after adding the iodine and glycogen content [a.u.] was calculated using ImageJ software (http://rsbweb.nih.gov/ij/) (access on 12 July 2018). Correlation between glycogen content (a.u.) and DNA content (a.u.) was considered. Correlation analysis of the data was performed using linear correlation (Pearson r) test.

### 3.8. FTIR Spectroscopy

Fourier-transform infrared (FTIR) spectroscopy measurements were performed using the Vertex 70 (Bruker, Poznan, Poland) spectrometer using the attenuated total reflectance (ATR) technique. The range of selected infrared radiation was the average IR (400–4000 cm^−1^). 32 scans with 2 cm^–1^ spectral resolution were performed. Normalization and baseline correction of obtained spectra were considered. All spectra were analyzed using OPUS software (Bruker, Poznan, Poland).

### 3.9. Deconvolution of Amide I region (1600–1700 cm^−1^)

The secondary protein structure was analyzed by means of curve fitting using MagicPlot 2.1. software (https://magicplot.com/downloads.php) (access on 3 July 2018). First, the secondary derivative spectra were determined based on the ATR-FTIR spectra to determine the initial peak positions for curve fitting, and the peaks were fitted using Gaussian function. The area under the curve was considered 100% and each component was expressed as its percentage after fitting.

### 3.10. Raman Spectroscopy

FT-Raman spectra were recorded using a Nicolet NXR 9650 FT-Raman Spectrometer equipped with an Nd:YAG laser (1064 nm) and a germanium detector. Measurements were performed in the range of 150 to 3700 cm^−1^ with a laser power of 1.5 W. An unfocused laser beam of a diameter of approximately 100 μm and a spectral resolution of 8 cm^−1^ was used. Raman spectra were processed by the Omnic/Thermo Scientific software based on 64 scans.

### 3.11. Lipid-Carbohydrate Ratio

To evaluate lipid-carbohydrate ratio, an area of peaks corresponding to lipid and carbohydrate vibrations were calculated [70]. The sum of the lipid as well as carbohydrate peak area were then calculated and the ratio of the sum of lipid and carbohydrate was calculated. To evaluate lipid-carbohydrate ratio, ORIGIN software was used.

### 3.12. Ergosterol Content

To estimate the levels of ergosterol, FTIR and Raman spectra of ergosterol were obtained. Ergosterol (Sigma-Aldrich, Poznan, Poland) was used as a reference standard. The value of intensity of individual peak from FTIR as well as Raman spectra at 1247 cm^−1^ and 1459 cm^−1^, respectively, was considered.

### 3.13. Multivariate Data Analysis

All obtained spectra were subjected to multivariate analysis using principal component analysis (PCA) and hierarchical cluster analysis (HCA) using PAST 3.0. software. HCA was based on Euclidean distance and Ward’s algorithms. The PCA and HCA were performed for all FTIR as well as Raman spectral ranges.

Moreover, a joined clustering analysis of chromosome number, DNA content, 11 vibrations of FTIR spectrum, seven vibrations of Raman spectrum, alpha-helix/beta-sheet ratio and lipid-carbohydrate ratio was performed using ClustVis, a web tool for visualizing clustering of multivariate data (BETA) (https://biit.cs.ut.ee/clustvis/) (access on 3 September 2018). [71]. Species clustering as well as antifungal treatment were included. A heat map was generated on the basis of karyotype profiling, DNA content analysis and signal intensities of some selected vibrations of FTIR and Raman spectra, namely FTIR vibrations: (1) C–O, C–O–H, and C–O–C deformation and C–C stretching vibrations from carbohydrates, (2) β(1–3) glucans, nucleic acids and glycogen, PO^2–^ symmetric stretching vibrations mainly from RNA, (3) C–O asymmetric stretching vibrations from phospholipids and lipids, (4) amide III: C–N and C–O stretching vibrations, N–H and O=C–N bending vibrations, (5) C=O of COO^−^ symmetric stretching vibrations from proteins, CH_2_ wagging vibrations from lipids and β(1–3) glucans, (6) Amide II: mainly C–N stretching vibrations and N–H bending vibrations, (7) amide I: mainly C=O stretching vibrations and contributions of N–H bending vibrations, (8) C=O stretching vibrations from lipid esters, (9) CH_2_ stretching vibrations from lipids, (10) CH_3_ stretching vibrations from lipids, (11) amide A from proteins; Raman vibrations: (1) symmetric benzene/pyrrole in-phase and out of phase breathing mode of tryptophan and phenylalanine, (2) symmetric benzene/pyrrole in-phase and out of phase breathing mode of tryptophan and phenylalanine, (3) C–H deformation vibrations from proteins, (4) C–H deformation vibrations from lipids, (5) ring stretching vibrations from the deoxyribonucleotide adenosine monophosphate, (6) amide I: mainly C=O stretching vibrations and contributions of N–H bending vibrations, and (7) C–H stretching vibration from lipids.

### 3.14. Statistical Analysis

The mean values ± SD were calculated on the basis of at least three independent experiments. Box and whisker plots were also considered. Statistical significance was evaluated using GraphPad Prism 5 using one-way ANOVA and Tukey’s test.

## Figures and Tables

**Table 1:** Clinical *Candida* species isolates used in the present study. Three reference strains, namely haploid, diploid, and tetraploid strains were also considered.

Isolate	Isolate Identification (API^®^ *Candida* Biochemical Test)	Isolate Identification (DNA Sequencing)	GenBank Accession Number	Sequence Identity (%)/Query Cover (%)	Isolation Site	Sex	Treatment
1.	*Candida albicans* 4316	*Candida albicans M366B*	KP675609.1	99/98	bronchoalveolar lavage	female	voriconazole
2.	*Candida albicans* 154	*Candida albicans NG76*	MH019247.1	99/96	vagina	female	
3.	*Candida albicans* 4200	*Candida glabrata*	AM492797.1	98/98	sputum	male	fluconazole
4.	*Candida albicans* 4248	*Candida albicans M215B*	KP675383.1	99/98	urine	female	
5.	*Candida albicans* 4310	*Candida albicans CGP41*	MF276783.1	99/100	sputum	female	
6.	*Candida albicans* M/529	*Candida albicans M179A*	KP675353.1	99/100	bronchoalveolar lavage	male	fluconazole
7.	*Candida albicans* 532	*Candida albicans M221B*	KP675393.1	99/98	bronchoalveolar lavage	male	
8.	*Candida albicans* 4331	*Candida albicans M363B*	KP675603.1	99/98	inoculation from urine	male	fluconazole
9.	*Candida albicans* 4324	*Candida albicans M366B*	KP675609.1	99/97	sputum	female	
10.	*Candida albicans* 534	*Candida sp.*	KY996547.1	99/97	bronchoalveolar lavage	male	
11.	*Candida albicans* 152	*Candida albicans M357B*	KP675591.1	99/99	vagina	female	
12.	*Candida albicans* 153	*Candida albicans H291B*	KP675000.1	89/97	vagina	female	
13.	*Candida albicans* 521	*Candida albicans 125A*	KP765018.1	96/97	pharynx	male	
14.	*Candida albicans* 4369	*Candida albicans H296B*	KP675010.1	100/99	wound	female	
15.	*Candida albicans* 556	*Candida albicans n96b*	KP675680.1	100/96	bronchoalveolar lavage	female	
16.	*Candida albicans* 563	*Candida albicans M349A*	KP675580.1	100/97	bronchoalveolar lavage	male	
17.	*Candida albicans* 4335	*Candida albicans H257B*	KP674940.1	88/97	bronchoalveolar lavage	female	
18.	*Candida albicans* 539	*Candida albicans H294A*	KP675005.1	100/98	bronchoalveolar lavage	male	
19.	*Candida albicans* 7363	*Candida albicans M215B*	KP675383.1	100/98	bronchoalveolar lavage	female	
20.	*Candida tropicalis* 4403	*Candida tropicalis H260C*	KP674945.1	99/98	sputum	male	
21.	*Candida tropicalis* 4114	*Candida tropicalis CTR817*	KX664669.1	99/98	bronchoalveolar lavage	male	
22.	*Candida glabrata* 520	*Candida glabrata IFM 64525*	LC317501.1	99/97	bronchoalveolar lavage	male	
23.	*Candida glabrata* 4570	*Candida glabrata M9*	LC389275.1	99/97	sputum	male	
24.	*Candida glabrata* 144	*Candida glabrata H160*	MF187244.1	99/97	vagina	female	
25.	*Candida glabrata* 4246	*Candida glabrata H160*	LC389261.1	99/97	urine	female	fluconazole
26.	*Candida albicans* 302	*Candida albicans H194B*	KP674872.1	100/98	reference strain—n		
27.	*Candida albicans* SC5314	*Candida albicans SC5314*	CP017630.1	99/98	reference strain—2n		
28.	*Candida albicans* T15	*Candida albicans B280A*	KP674535.1	99/97	reference strain—4n		

## Supplementary Materials

Supplementary materials can be found at https://www.mdpi.com/1422-0067/20/4/988/s1. Figure S1. FTIR spectra of clinical *Candida* isolates and reference strains (1–28). Figure S2. Raman spectra of clinical *Candida* isolates and reference strains (1–28). Figure S3. Spectra of component bands of amide I FTIR region of clinical *Candida* isolates and reference strains (1–28). Table S1. The peak positions and comments on vibrations for clinical *Candida* isolates and reference strains based on both (a) FTIR spectroscopy and (b) Raman spectroscopy. Table S2. Analysis of secondary protein structures of clinical *Candida* isolates based on IR region between 1700–1600 cm^−1^ (amide I region). Table S3. Lipid-carbohydrate ratio based on FTIR spectroscopy. Table S4. FTIR spectroscopy –peak area for lipid-carbohydrate ratio of clinical *Candida* isolates and reference strains (1–28).
