# Supplementary material for: FTIR and Raman Spectroscopy-Based Biochemical Profiling Reflects Genomic Diversity of Clinical Candida Isolates That May Be Useful for Diagnosis and Targeted Therapy of Candidiasis
Source: Int J Mol Sci. 2019 Feb 25;20(4):988. doi: 10.3390/ijms20040988 (PMC6412866; doi:10.3390/ijms20040988)
Supplement: Supplementary file 1 [file ijms-20-00988-s001.zip › ijms-439484-supplementary (english edits)/Nowy folder/Supplemental Figure 3.pdf]

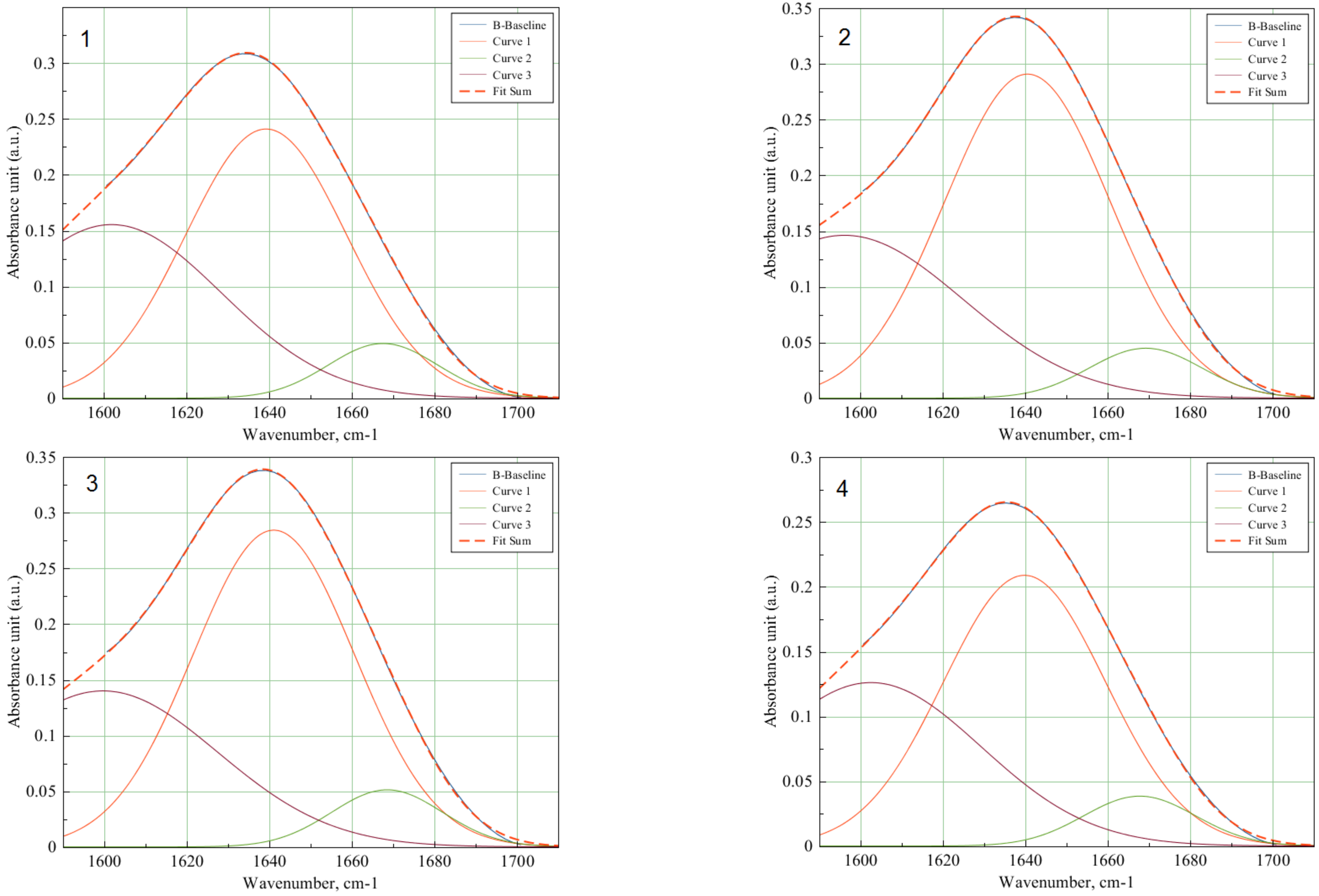

1
2
3
4
B-Baseline
Curve 1
Curve 2
Curve 3
Fit Sum
Absorbance unit (a.u.)
Wavenumber, cm-1

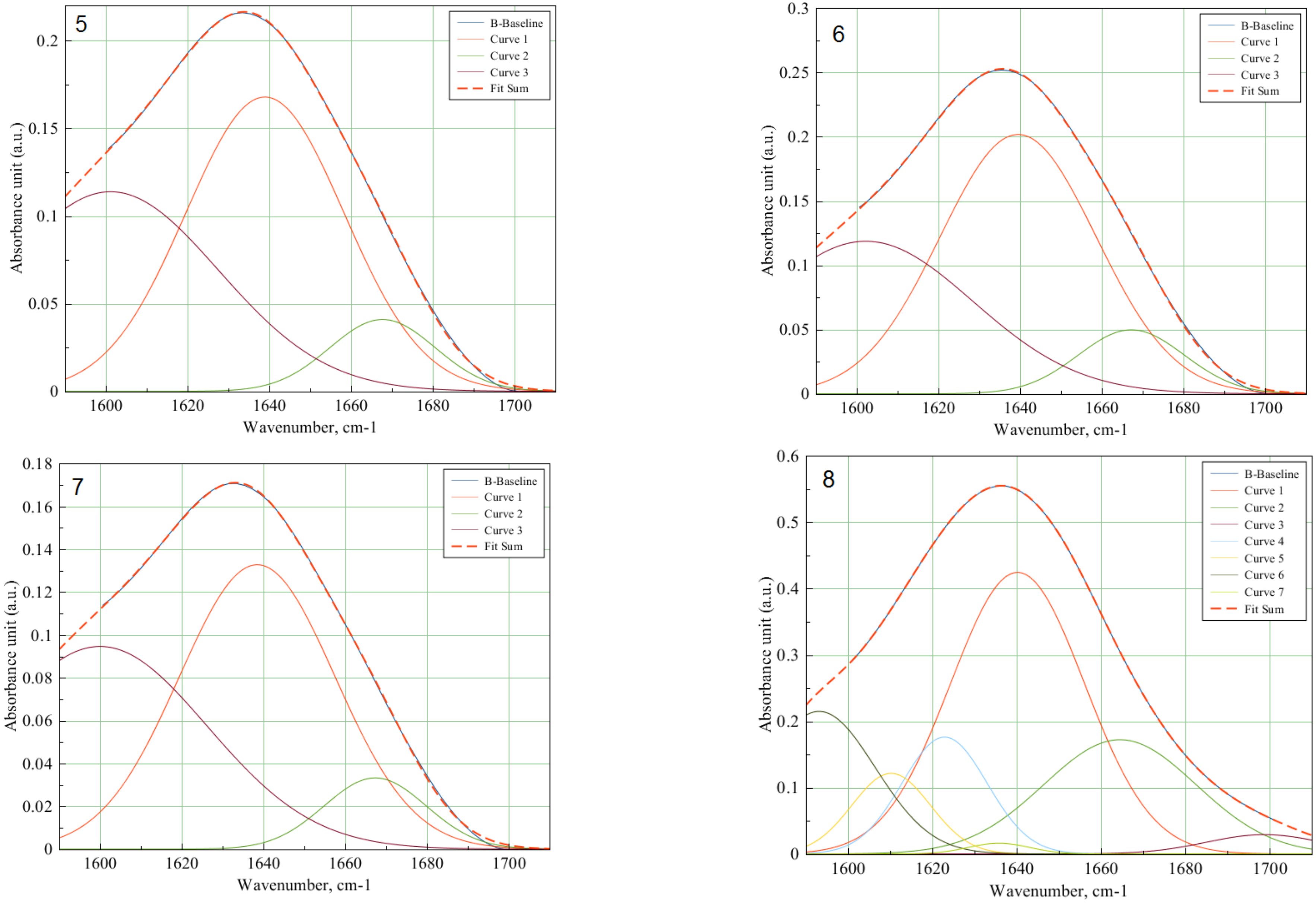
5
6
7
8
B-Baseline
Curve 1
Curve 2
Curve 3
Curve 4
Curve 5
Curve 6
Curve 7
Fit Sum
Absorbance unit (a.u.)
Wavenumber, cm-1
1600
1620
1640
1660
1680
1700

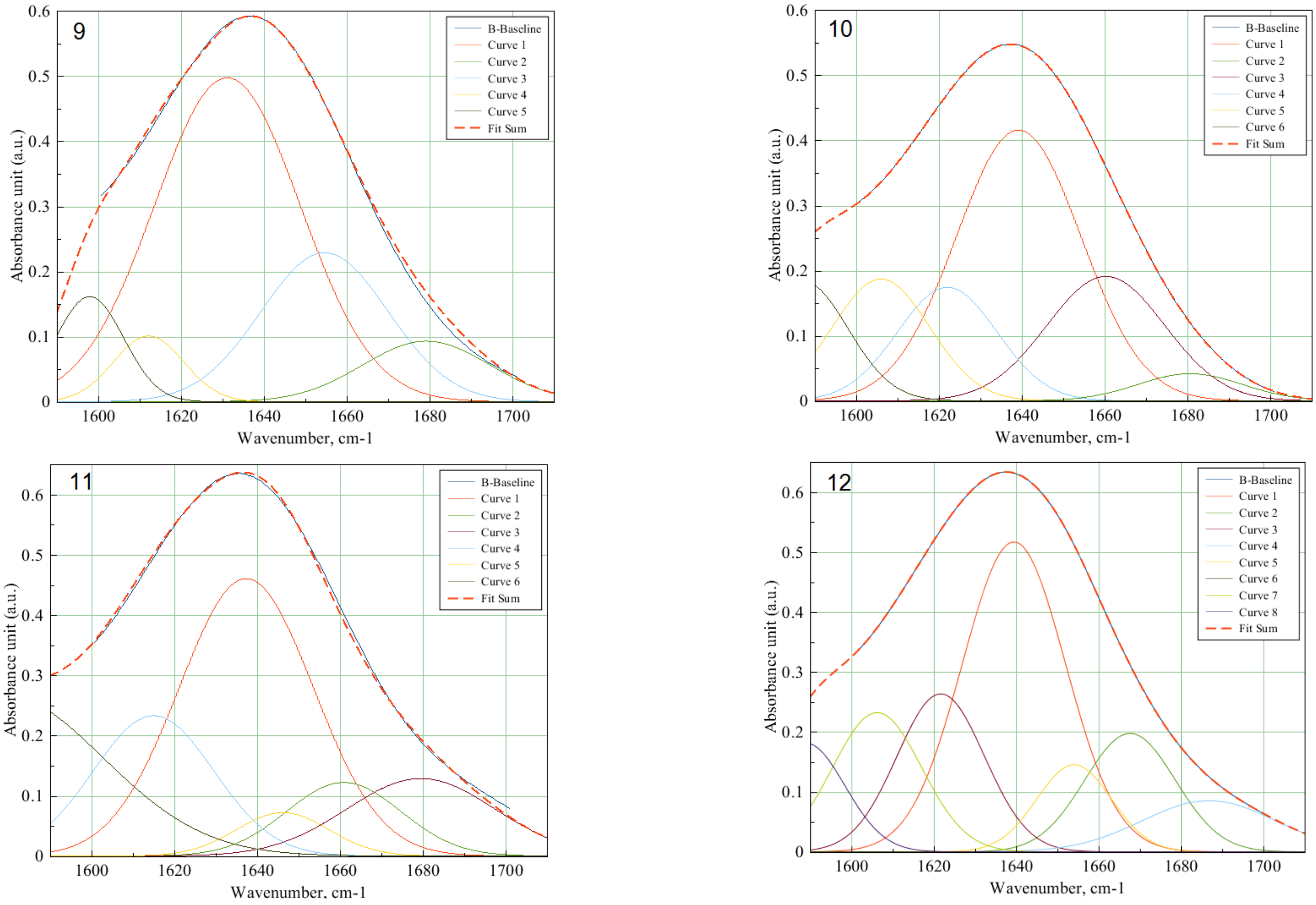
9
10
11
12
Absorbance unit (a.u.)
Wavenumber, cm-1
B-Baseline
Curve 1
Curve 2
Curve 3
Curve 4
Curve 5
Curve 6
Curve 7
Curve 8
Fit Sum
0
0.1
0.2
0.3
0.4
0.5
0.6
1600
1620
1640
1660
1680
1700

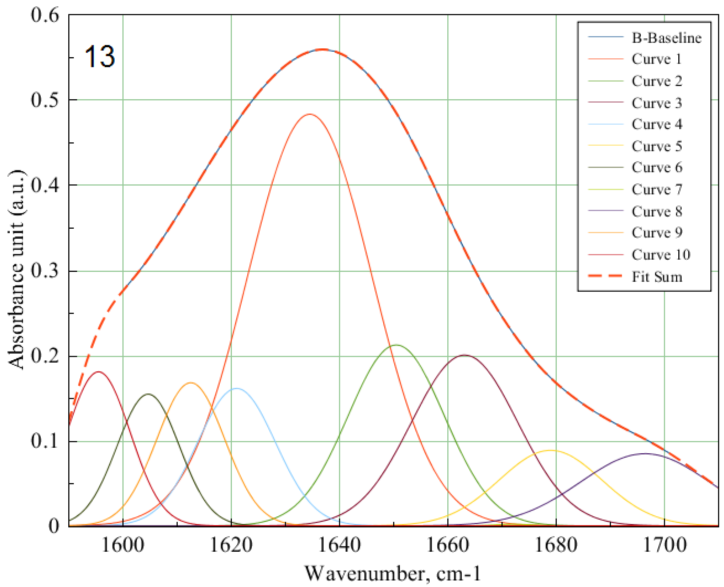
13
Absorbance unit (a.u.)
Wavenumber, cm-1
0 0.1 0.2 0.3 0.4 0.5 0.6
1600 1620 1640 1660 1680 1700
B-Baseline
Curve 1
Curve 2
Curve 3
Curve 4
Curve 5
Curve 6
Curve 7
Curve 8
Curve 9
Curve 10
Fit Sum

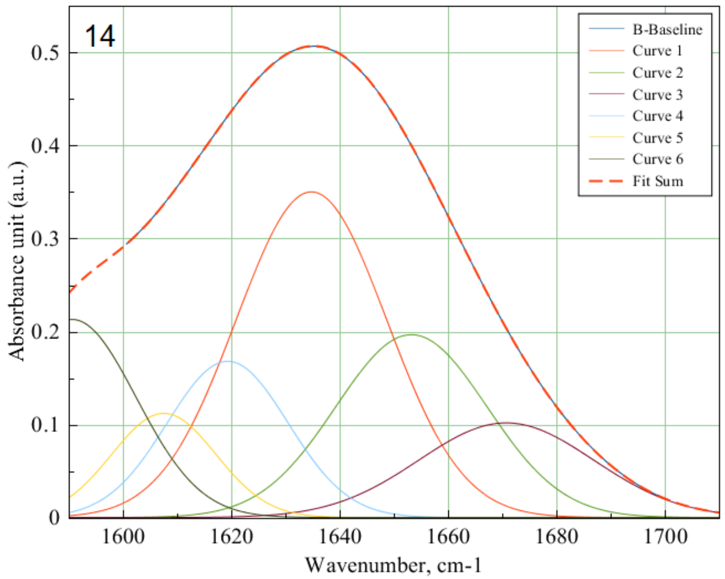
14
Absorbance unit (a.u.)
Wavenumber, cm-1
0 0.1 0.2 0.3 0.4 0.5
1600 1620 1640 1660 1680 1700
B-Baseline
Curve 1
Curve 2
Curve 3
Curve 4
Curve 5
Curve 6
Fit Sum

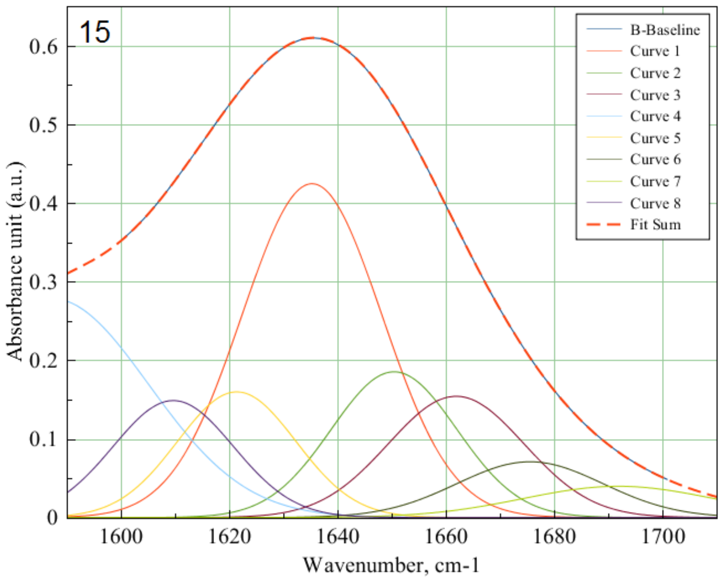
15
Absorbance unit (a.u.)
Wavenumber, cm-1
0 0.1 0.2 0.3 0.4 0.5 0.6
1600 1620 1640 1660 1680 1700
B-Baseline
Curve 1
Curve 2
Curve 3
Curve 4
Curve 5
Curve 6
Curve 7
Curve 8
Fit Sum

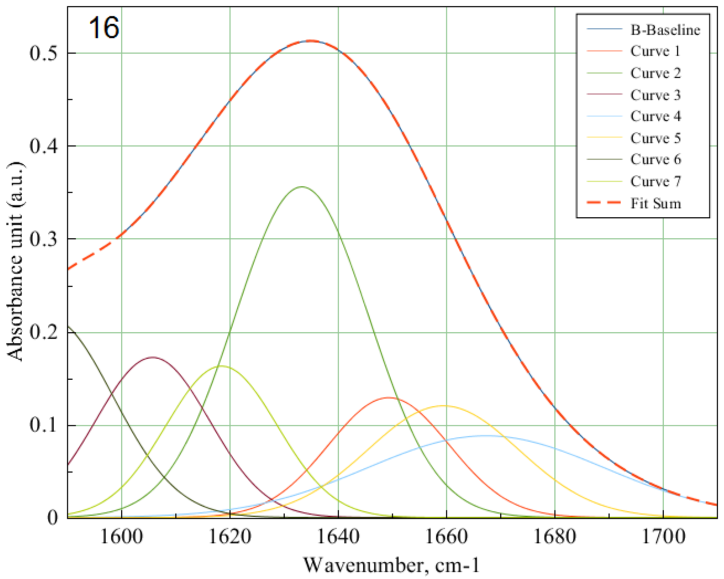
16
Absorbance unit (a.u.)
Wavenumber, cm-1
0 0.1 0.2 0.3 0.4 0.5
1600 1620 1640 1660 1680 1700
B-Baseline
Curve 1
Curve 2
Curve 3
Curve 4
Curve 5
Curve 6
Curve 7
Fit Sum

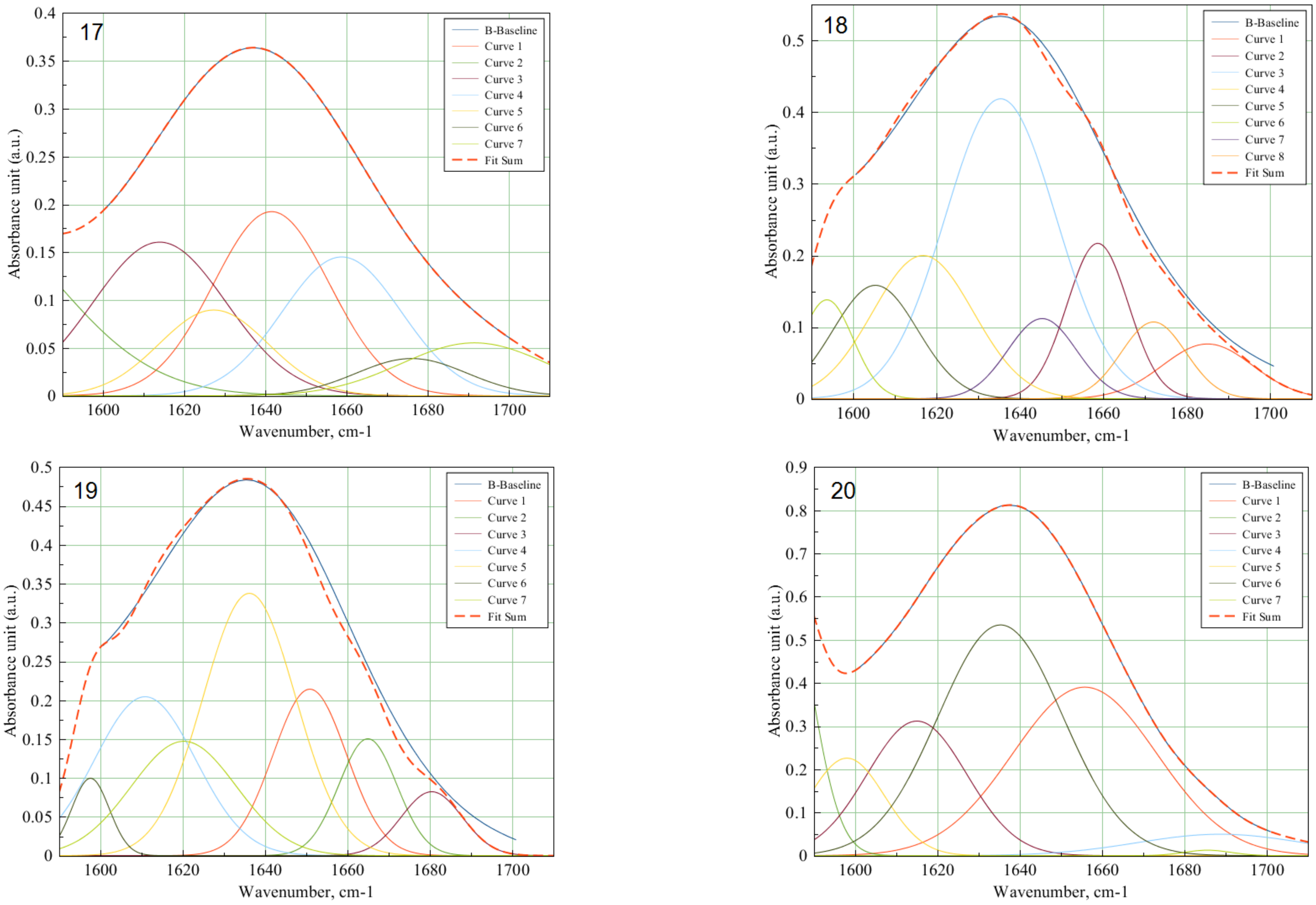
17
18
19
20
Absorbance unit (a.u.)
Wavenumber, cm-1
B-Baseline
Curve 1
Curve 2
Curve 3
Curve 4
Curve 5
Curve 6
Curve 7
Curve 8
Fit Sum

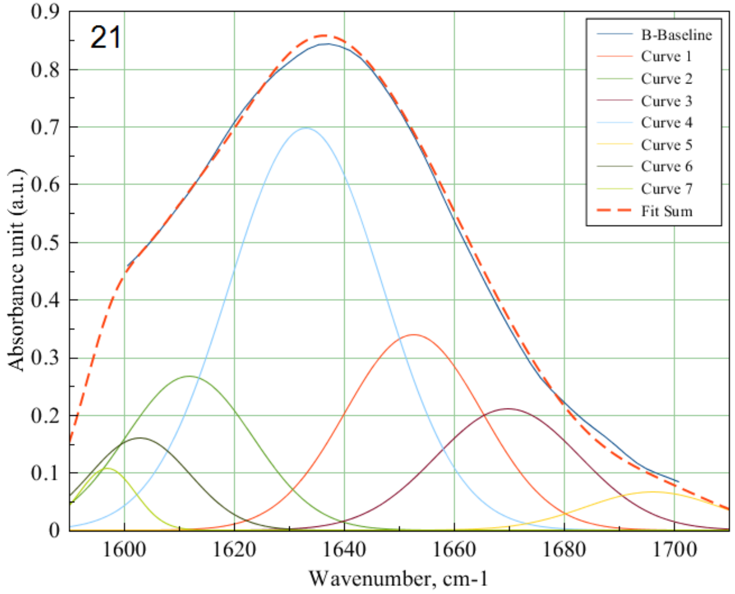
21
Absorbance unit (a.u.)
Wavenumber, cm-1
0.9 0.8 0.7 0.6 0.5 0.4 0.3 0.2 0.1 0
1600 1620 1640 1660 1680 1700
B-Baseline
Curve 1
Curve 2
Curve 3
Curve 4
Curve 5
Curve 6
Curve 7
Fit Sum

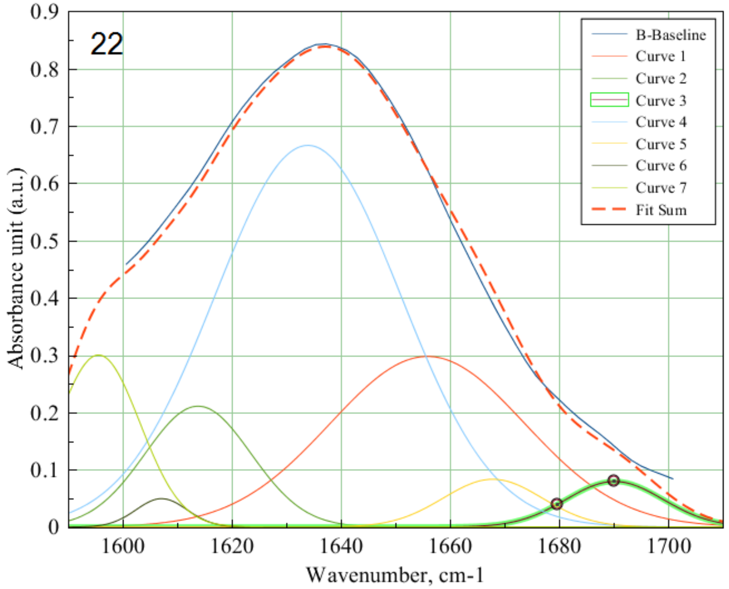
22
Absorbance unit (a.u.)
Wavenumber, cm-1
0.9 0.8 0.7 0.6 0.5 0.4 0.3 0.2 0.1 0
1600 1620 1640 1660 1680 1700
B-Baseline
Curve 1
Curve 2
Curve 3
Curve 4
Curve 5
Curve 6
Curve 7
Fit Sum

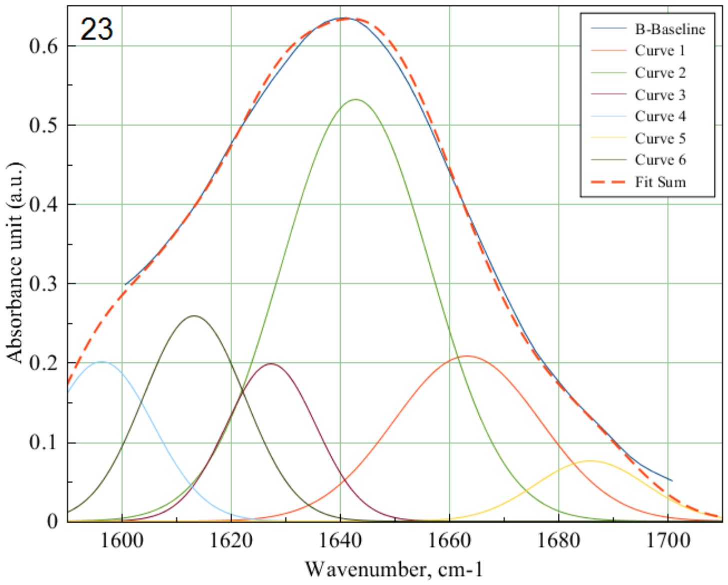
23
Absorbance unit (a.u.)
Wavenumber, cm-1
0.6 0.5 0.4 0.3 0.2 0.1 0
1600 1620 1640 1660 1680 1700
B-Baseline
Curve 1
Curve 2
Curve 3
Curve 4
Curve 5
Curve 6
Fit Sum

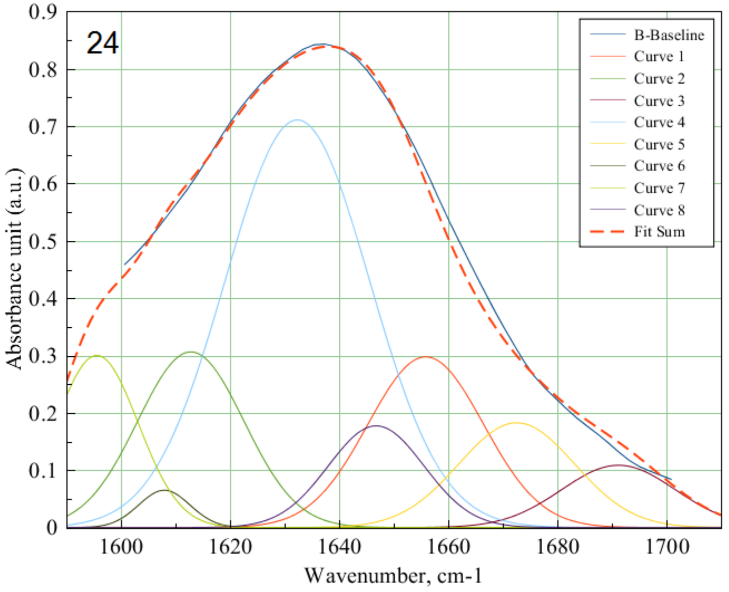
24
Absorbance unit (a.u.)
Wavenumber, cm-1
0.9 0.8 0.7 0.6 0.5 0.4 0.3 0.2 0.1 0
1600 1620 1640 1660 1680 1700
B-Baseline
Curve 1
Curve 2
Curve 3
Curve 4
Curve 5
Curve 6
Curve 7
Curve 8
Fit Sum

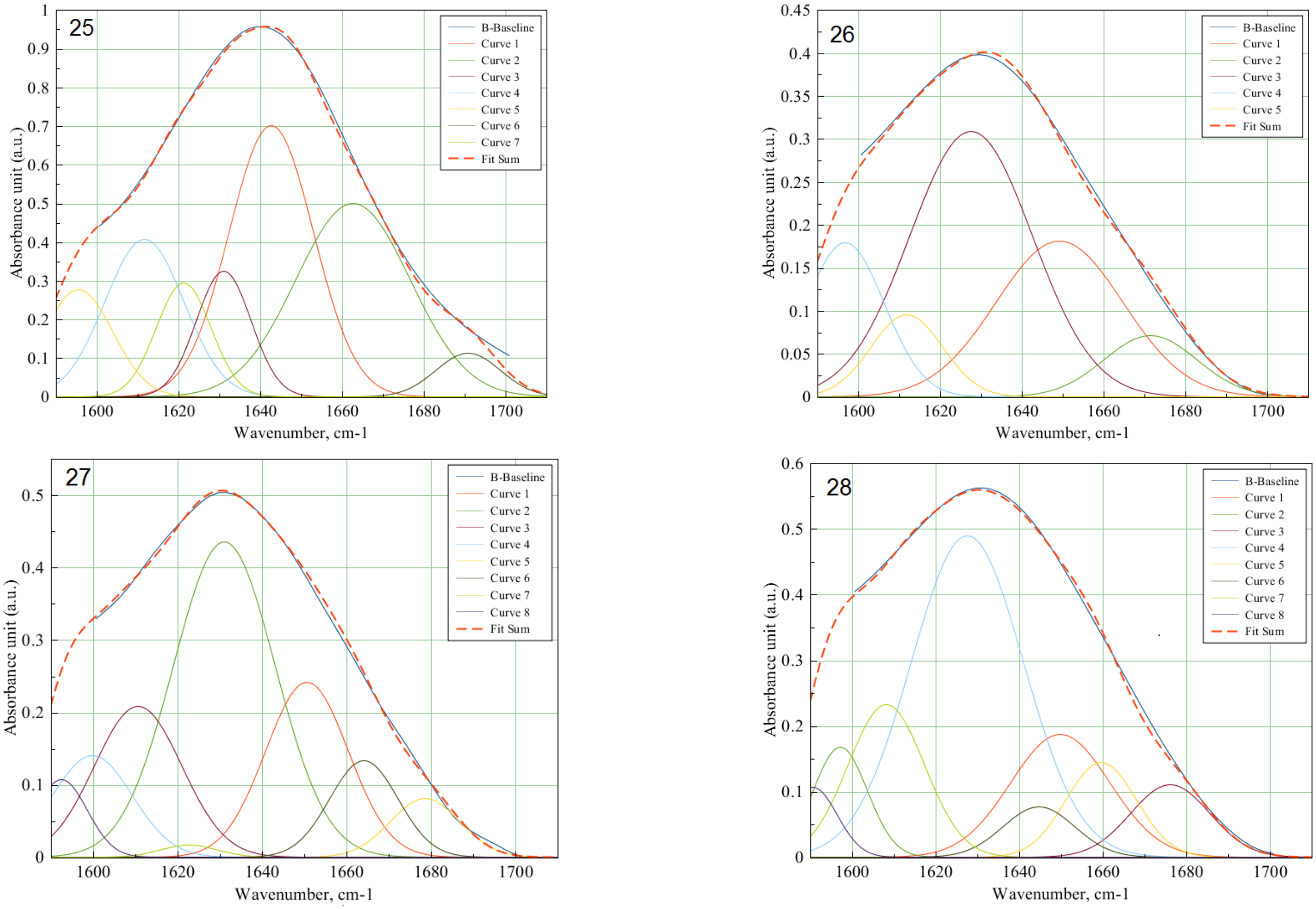

Supplemental Figure 3. Spectra of component bands of amide I FTIR region of clinical *Candida* isolates and reference strains (1-28).
