# Supplementary figures and images for: FTIR and Raman Spectroscopy-Based Biochemical Profiling Reflects Genomic Diversity of Clinical Candida Isolates That May Be Useful for Diagnosis and Targeted Therapy of Candidiasis
Source: Int J Mol Sci. 2019 Feb 25;20(4):988. doi: 10.3390/ijms20040988 (PMC6412866; doi:10.3390/ijms20040988)

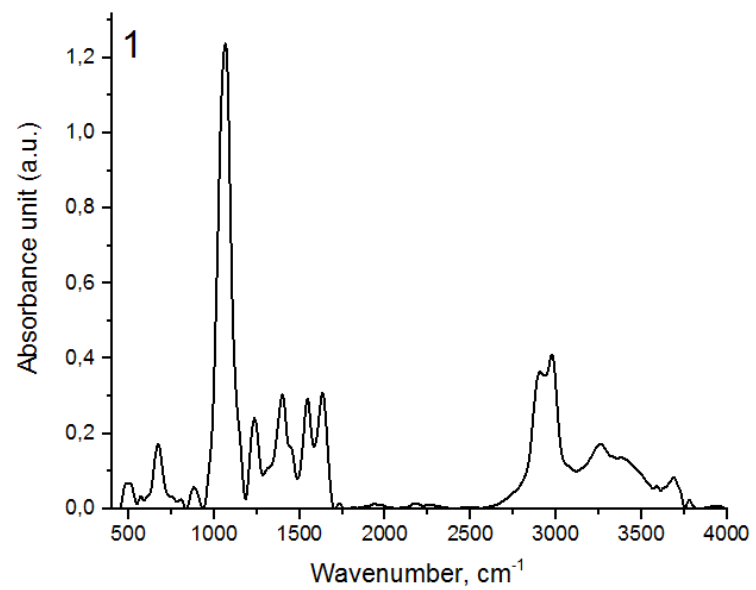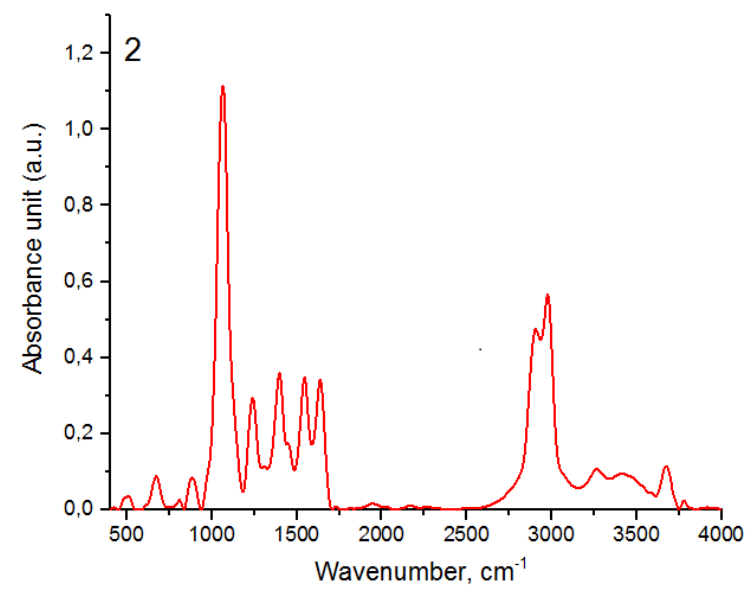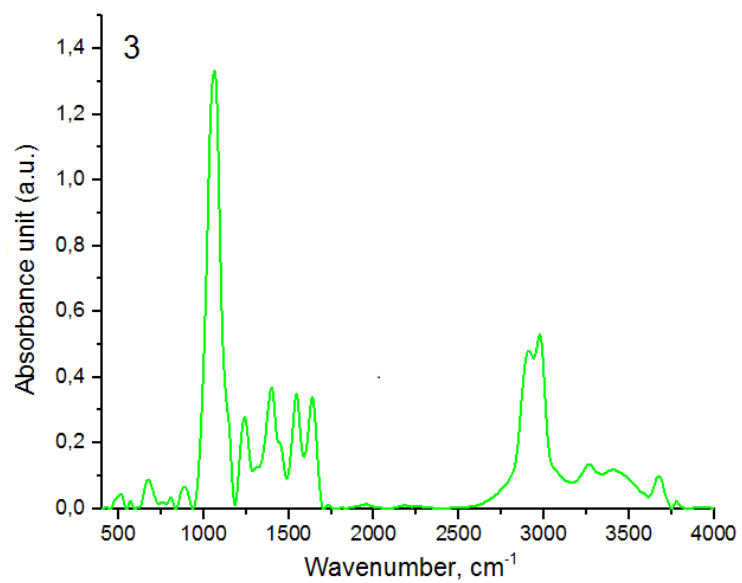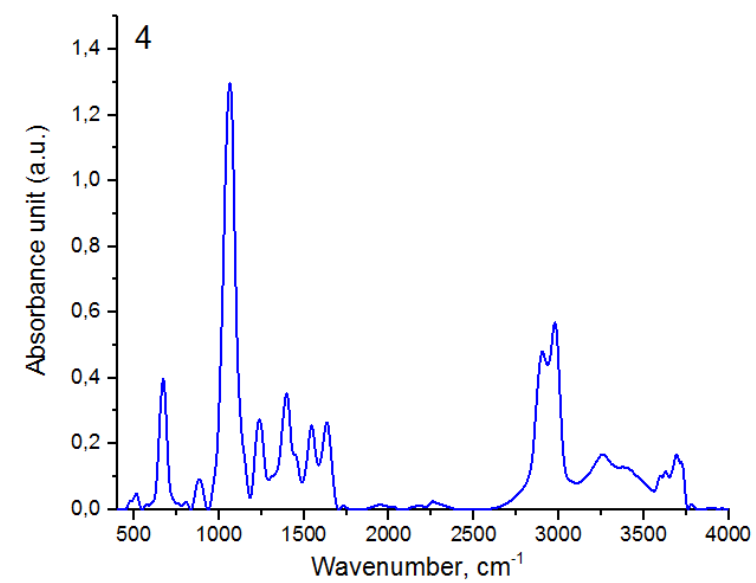

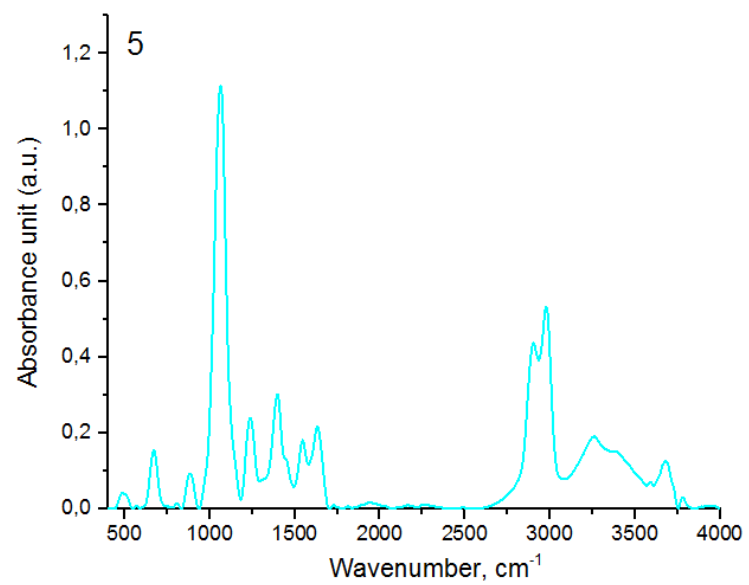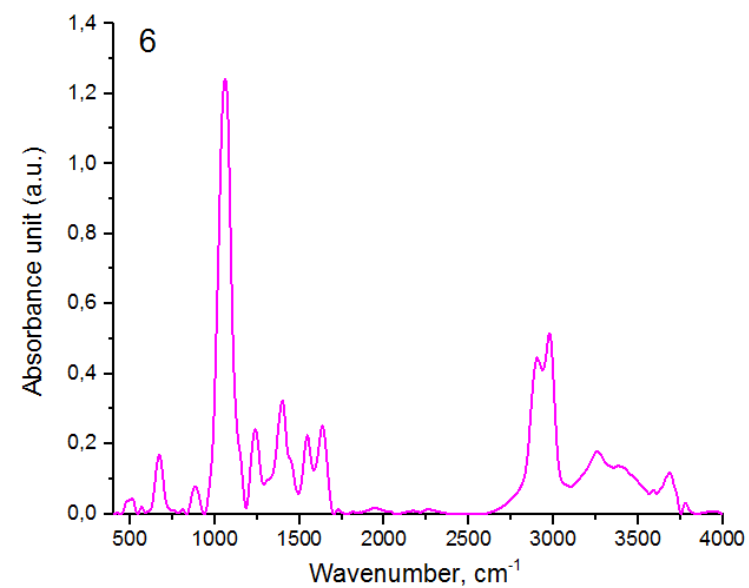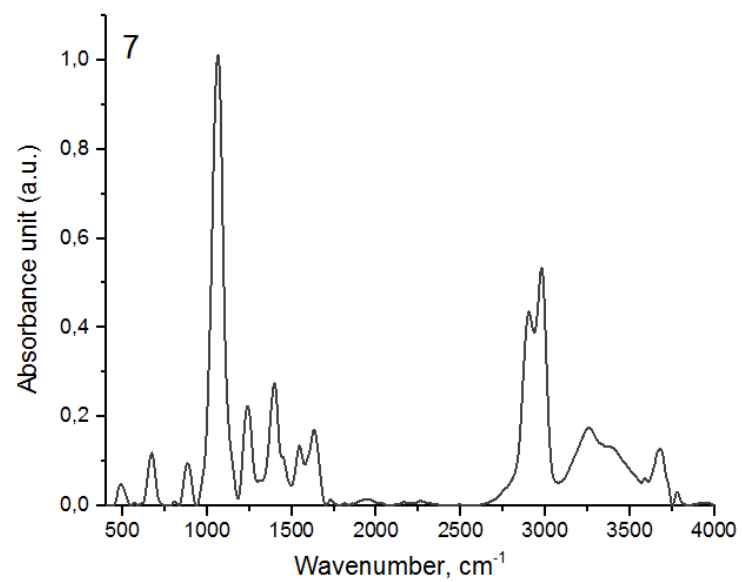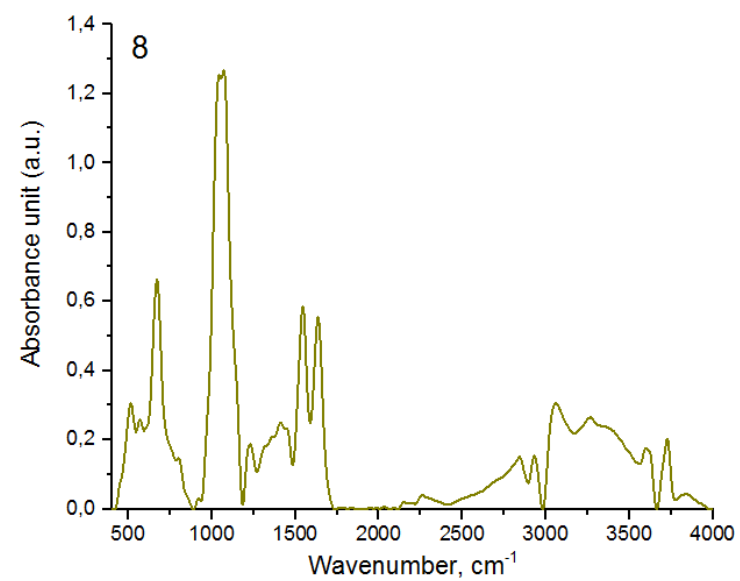

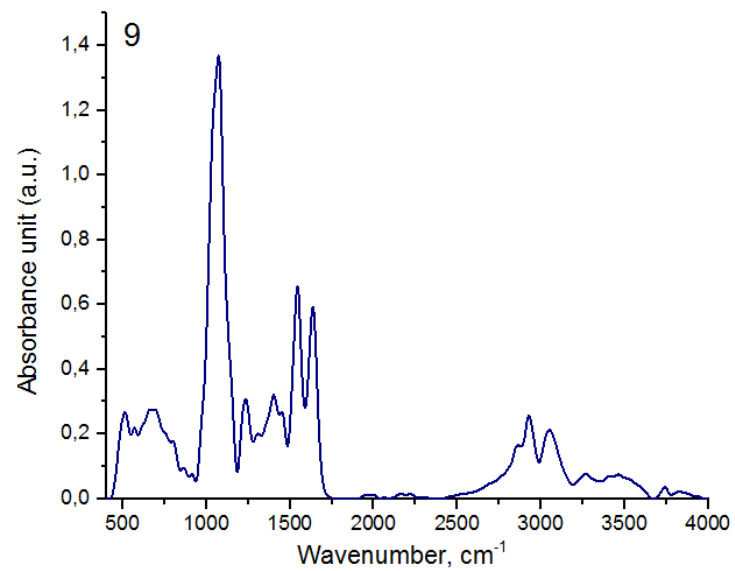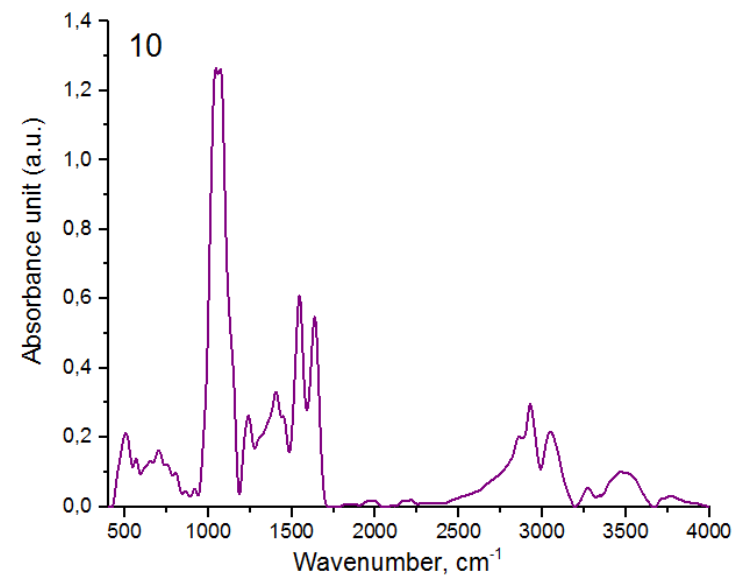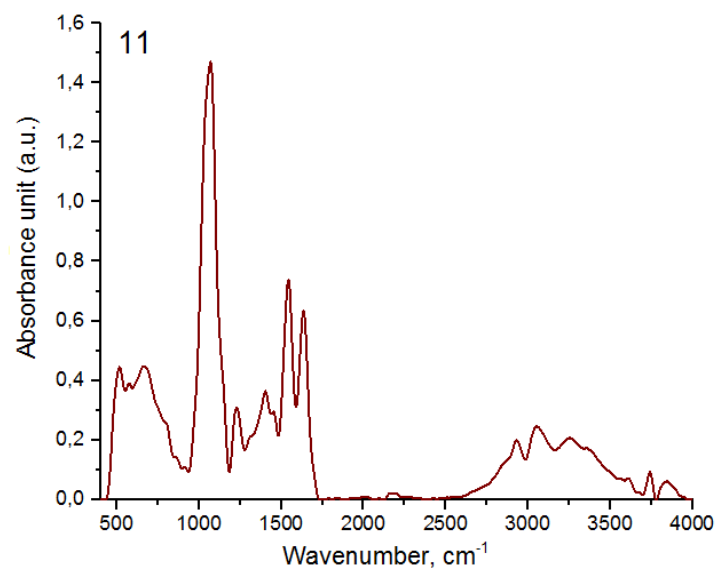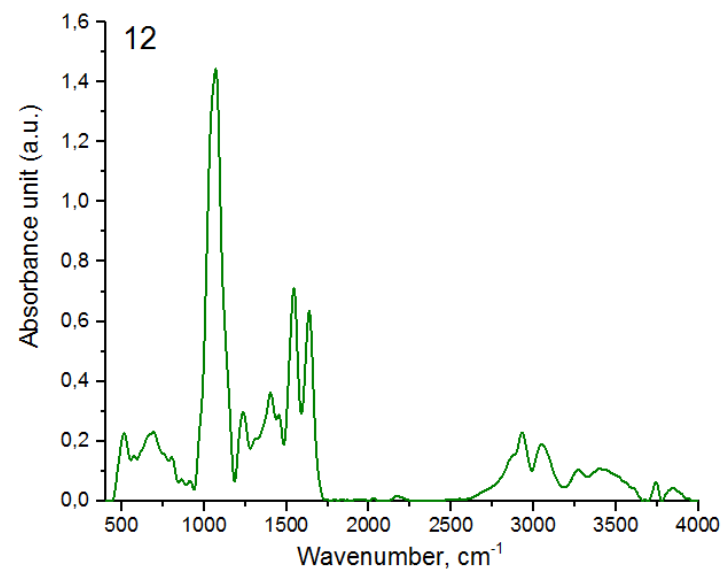

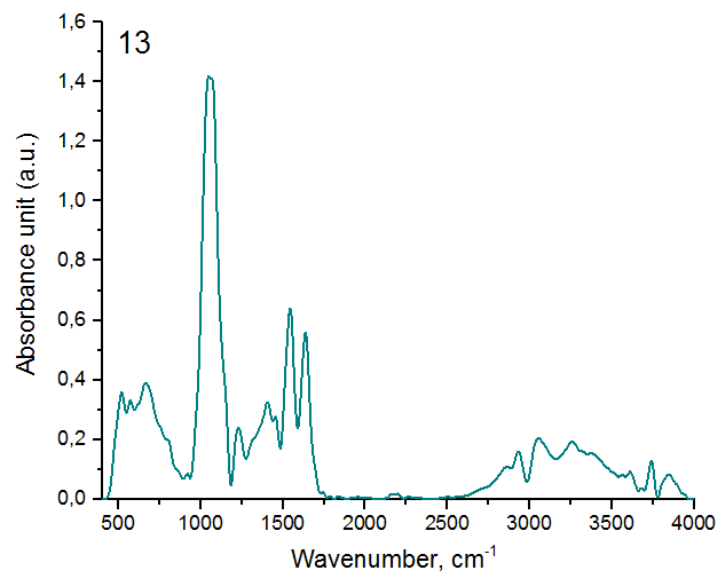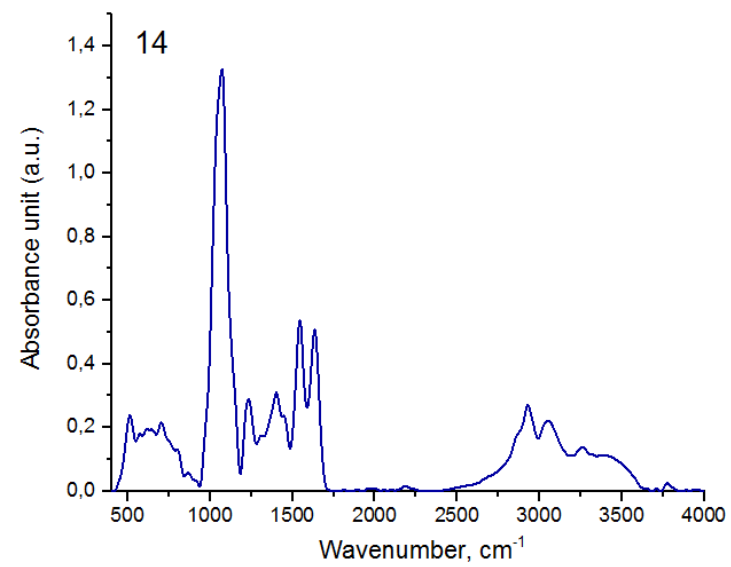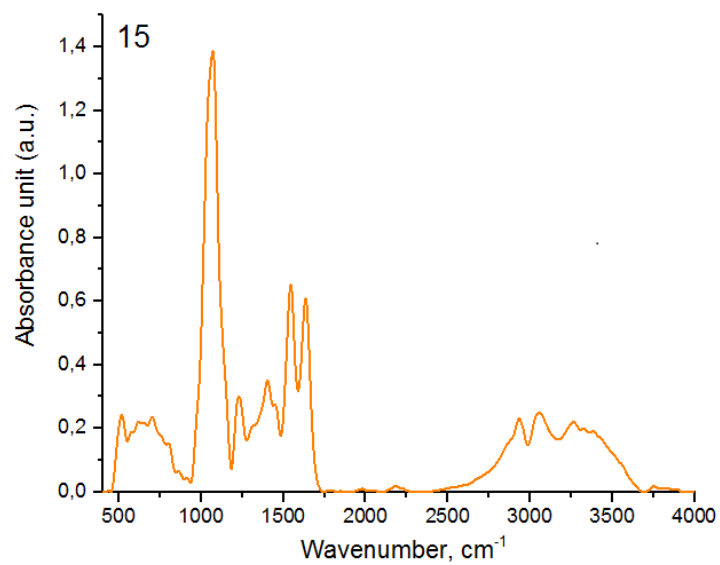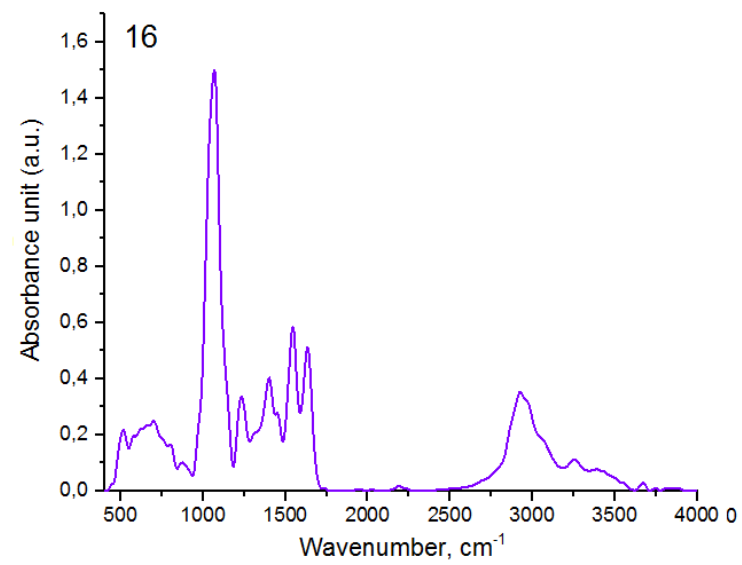

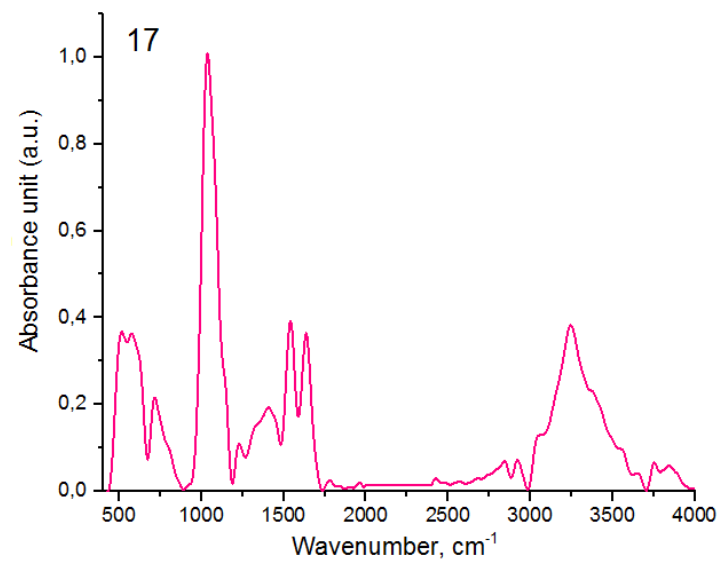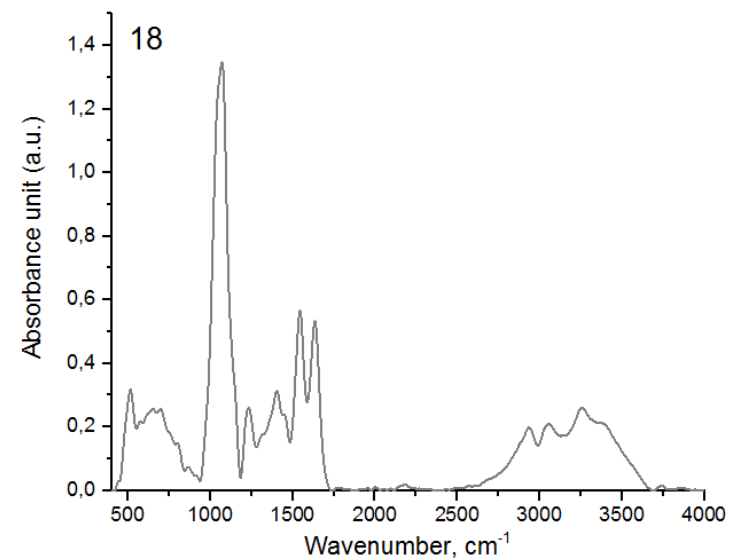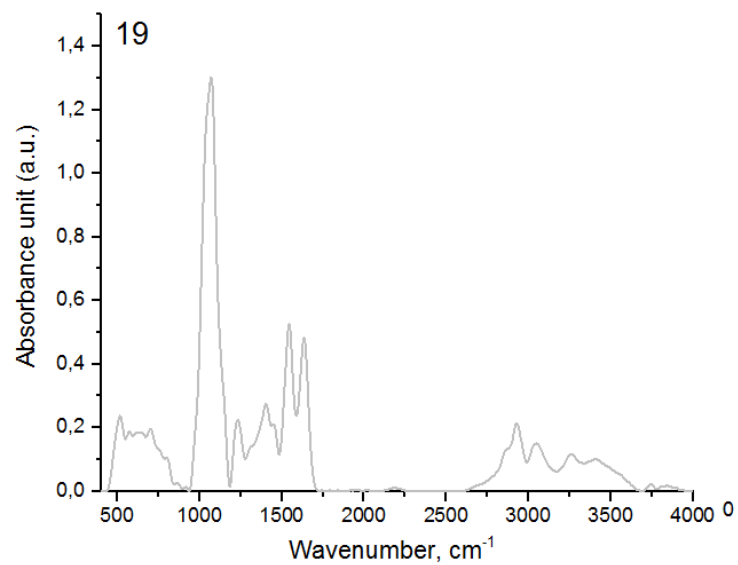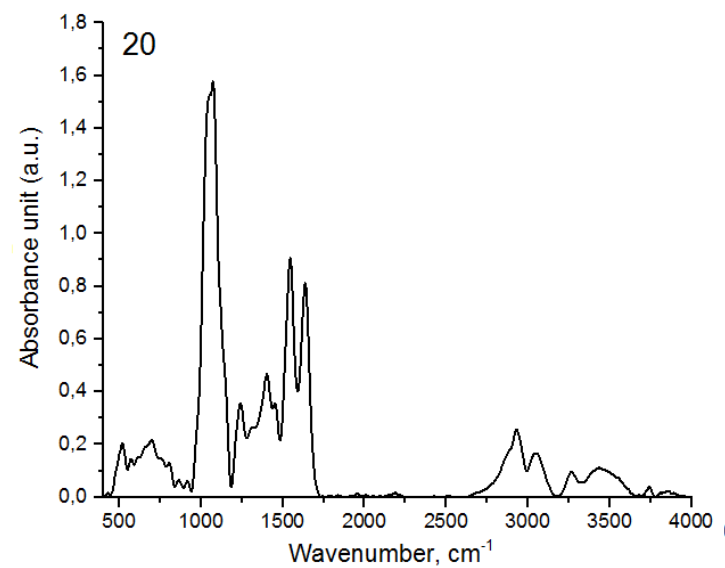

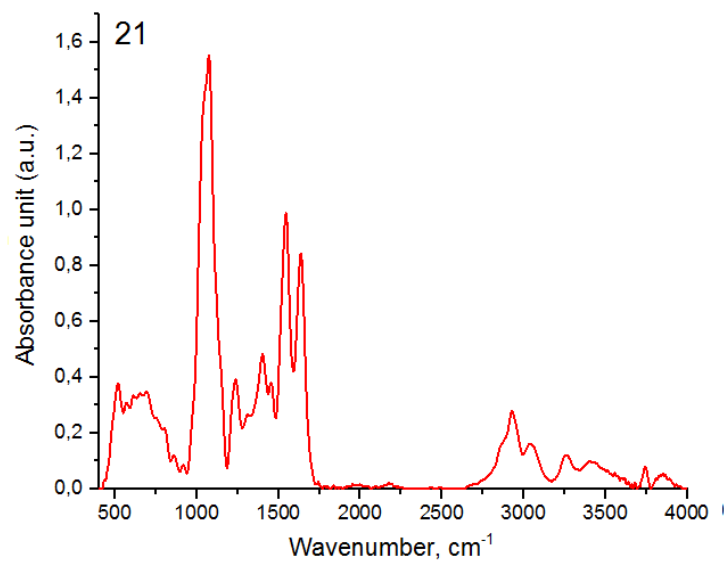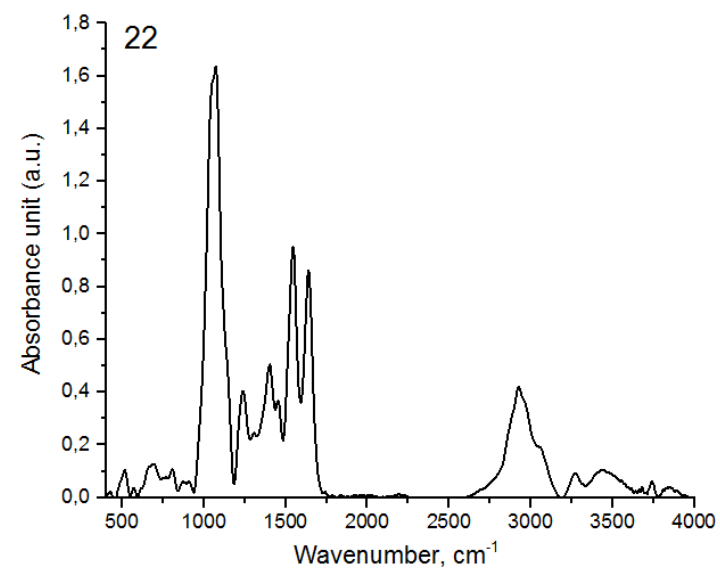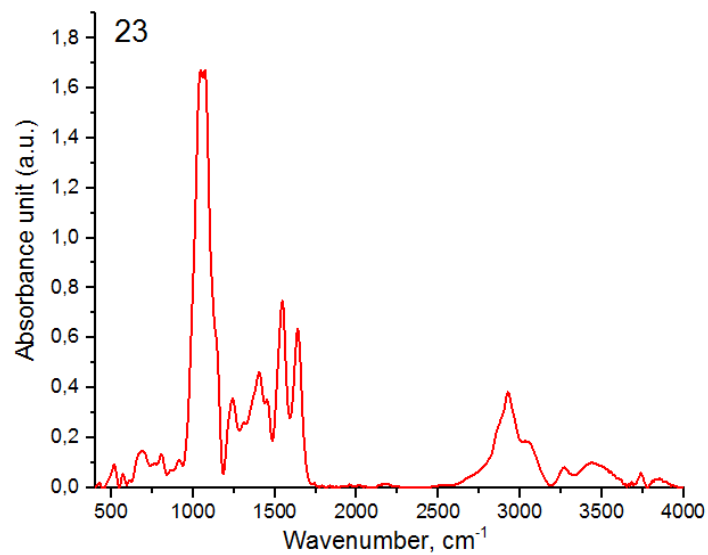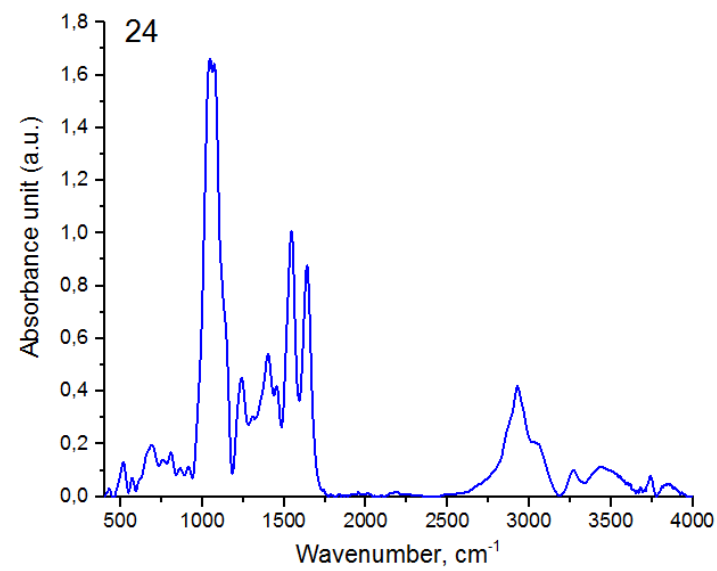

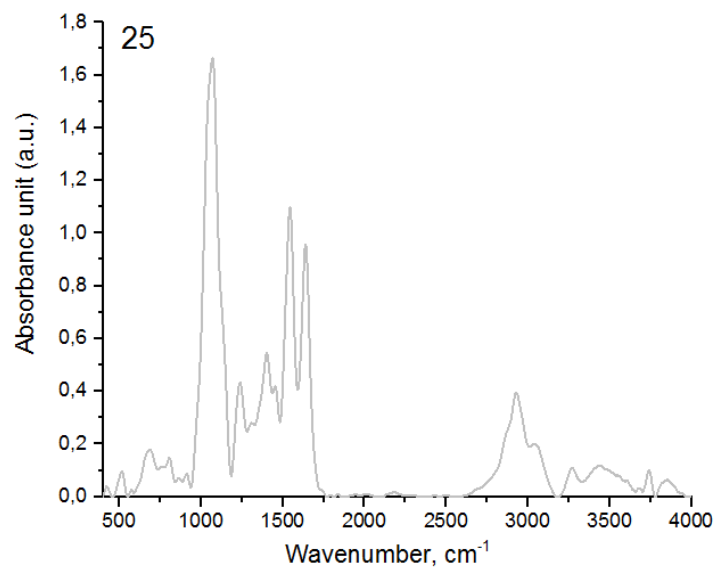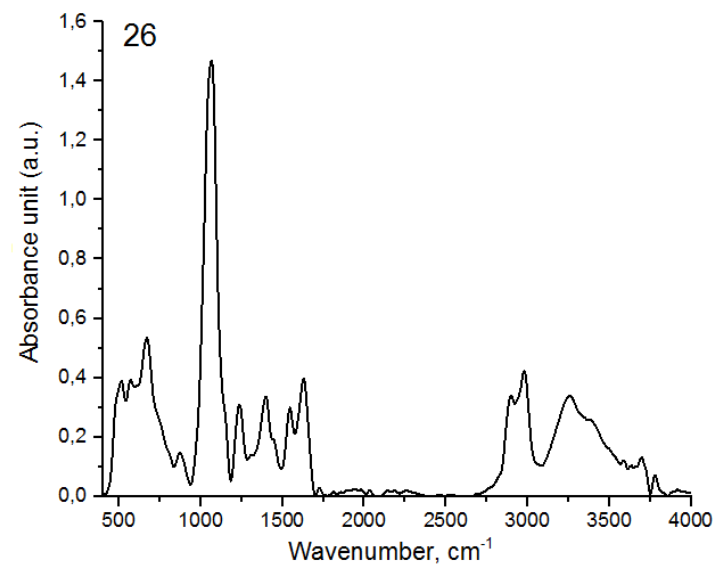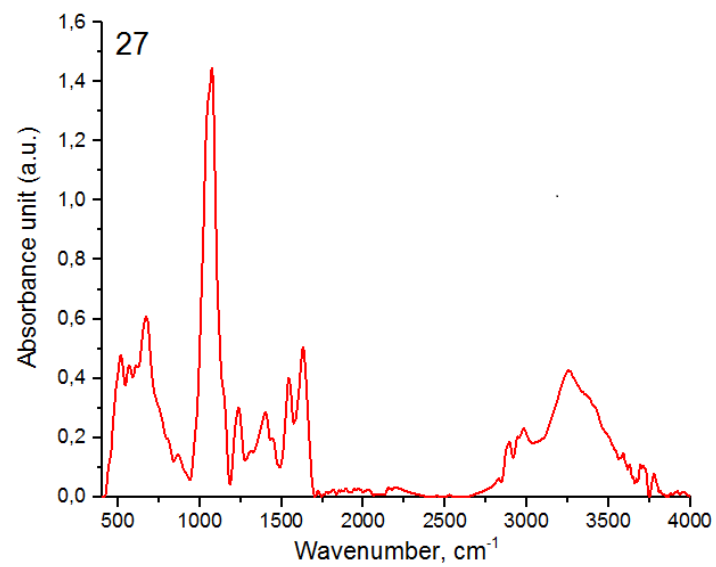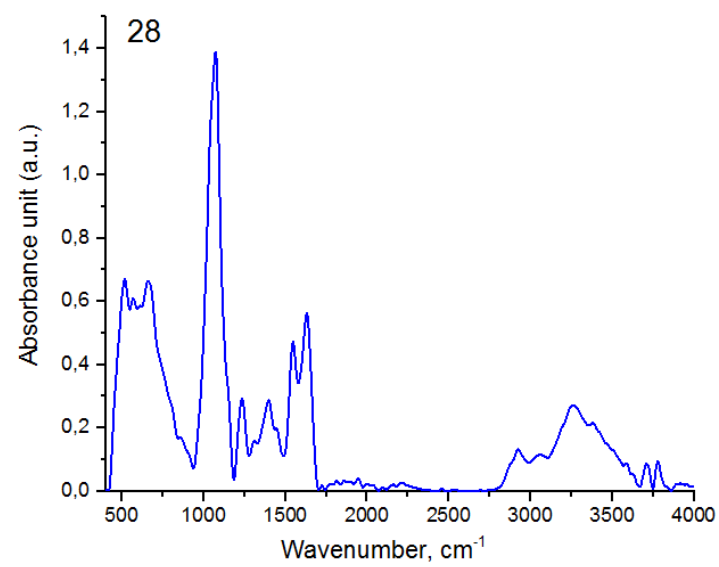

**Supplemental Figure 1.** FTIR spectra of clinical *Candida* isolates and reference strains (1-28).

Supplement: Supplementary file 1 [file ijms-20-00988-s001.zip › ijms-439484-supplementary (english edits)/Nowy folder/Supplemental Figure 1.pdf]

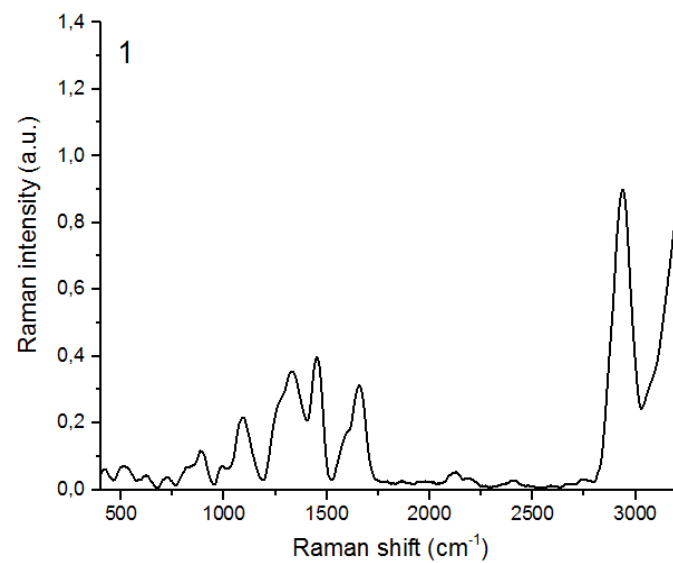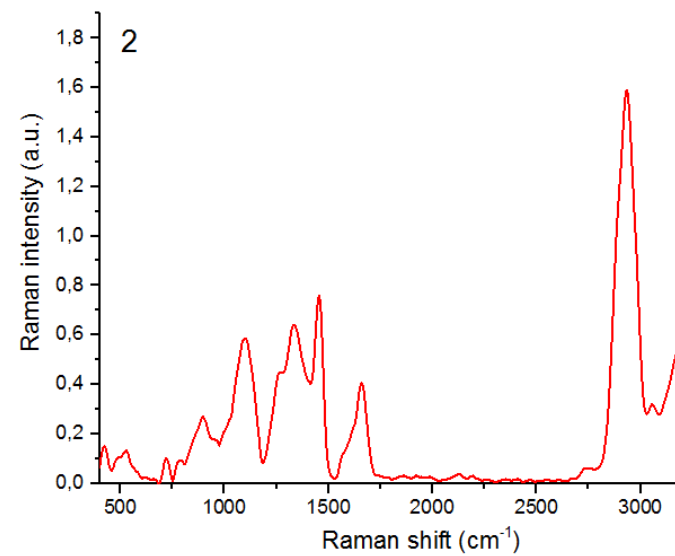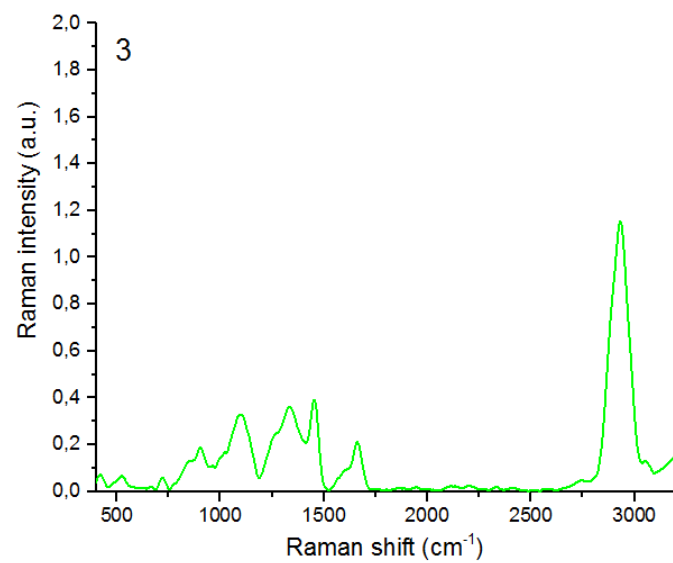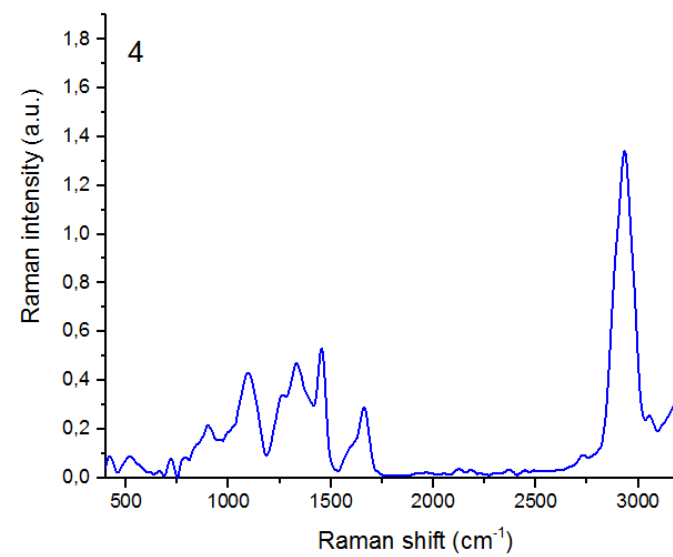

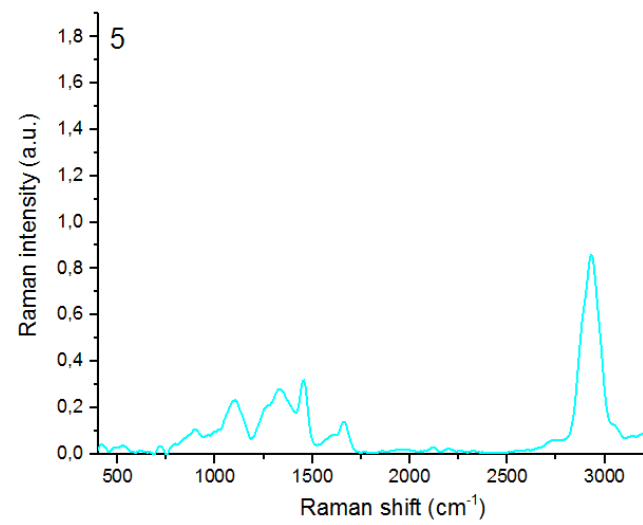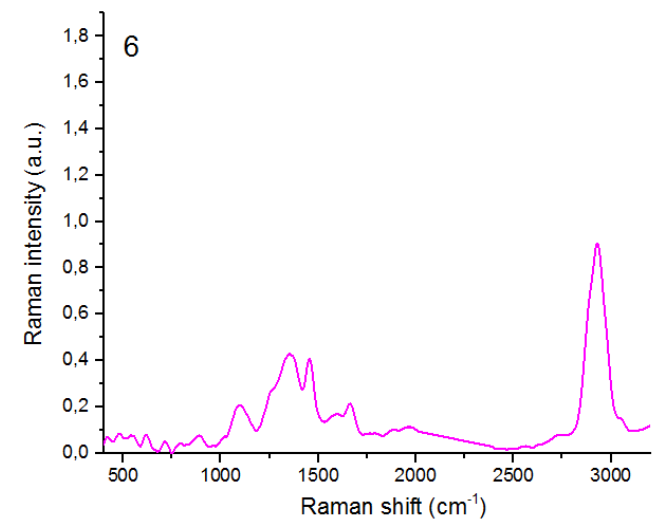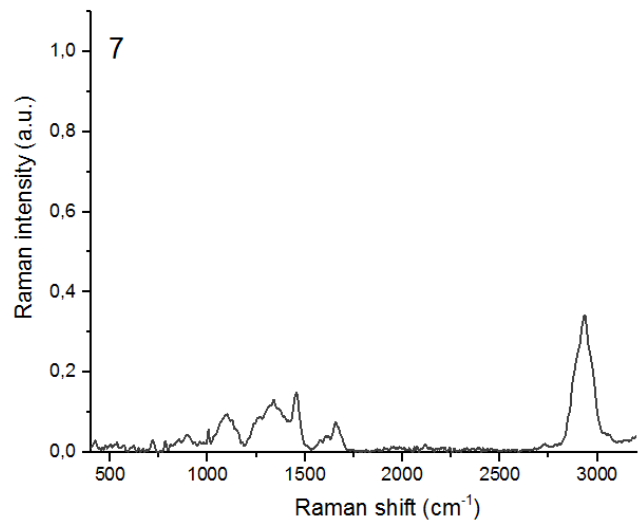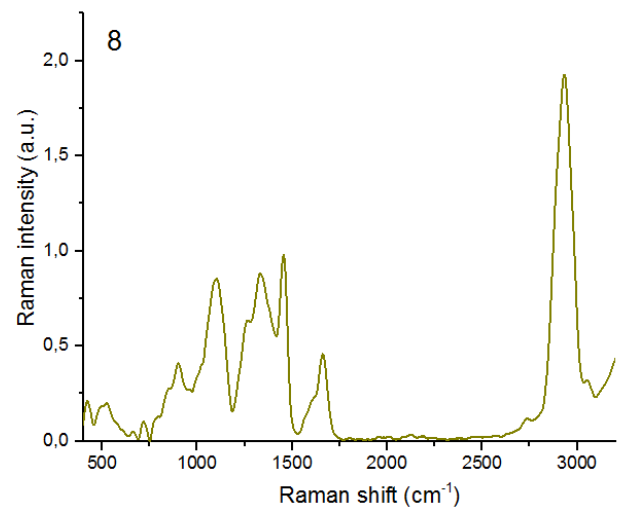

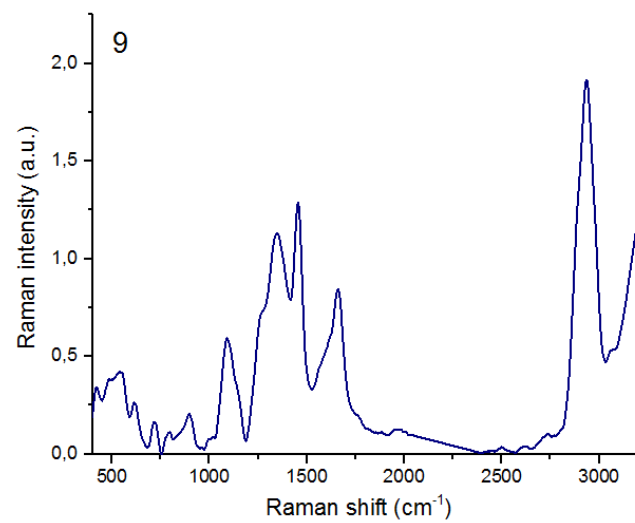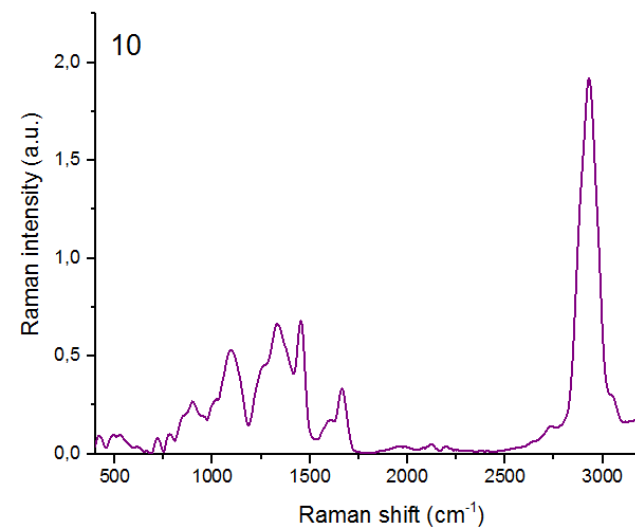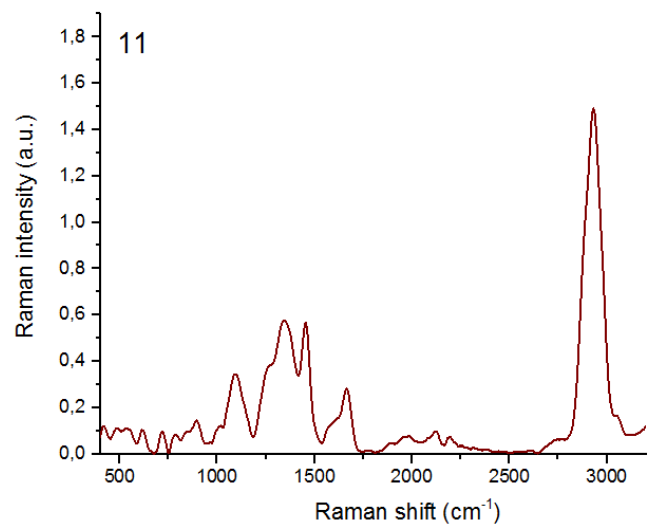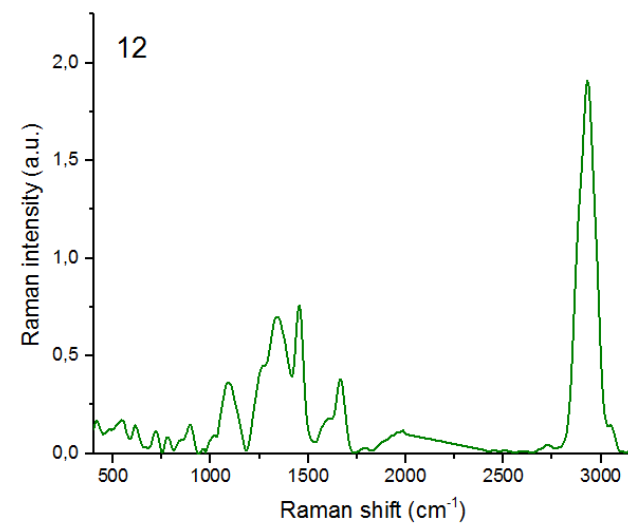

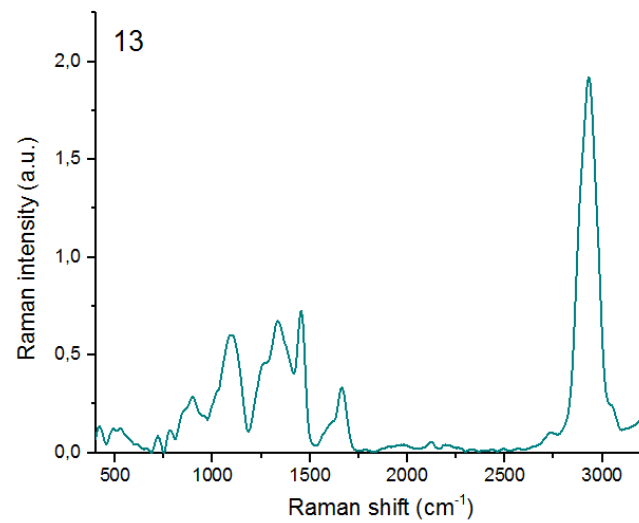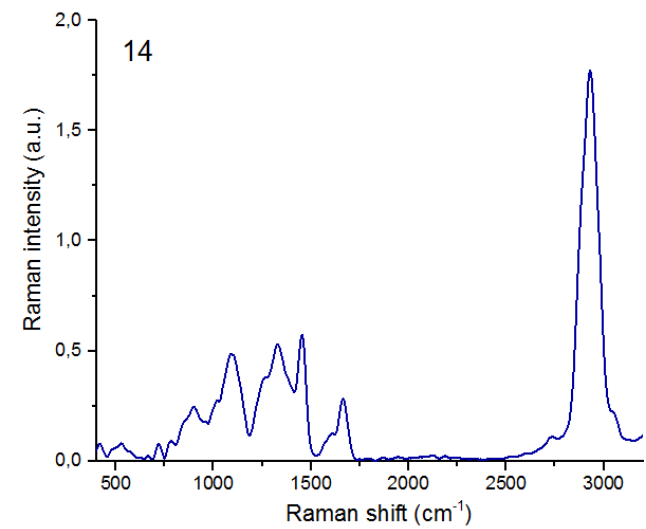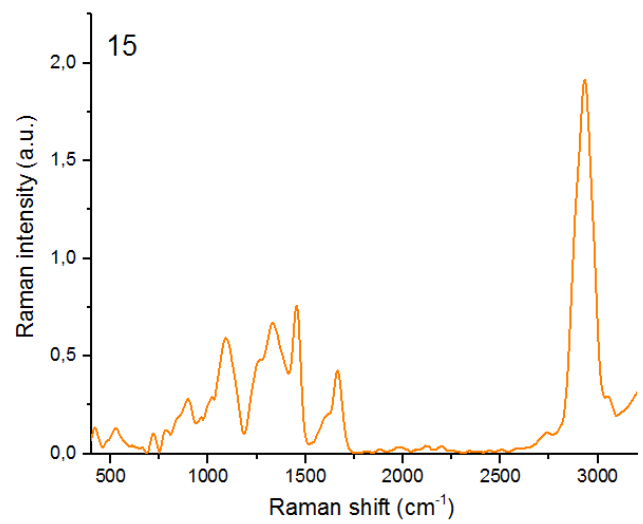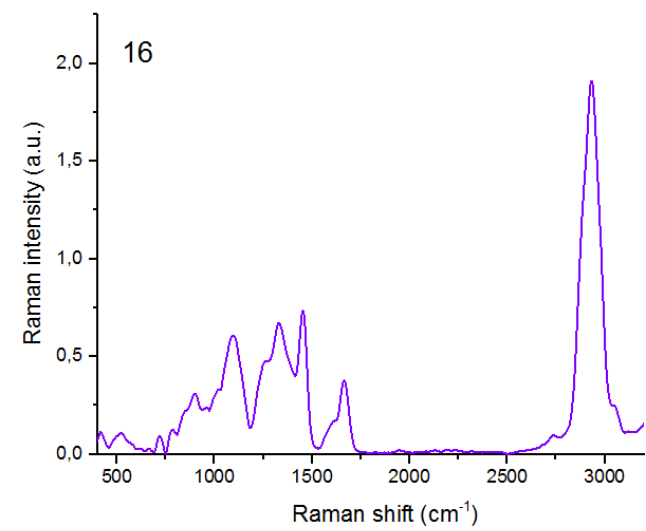

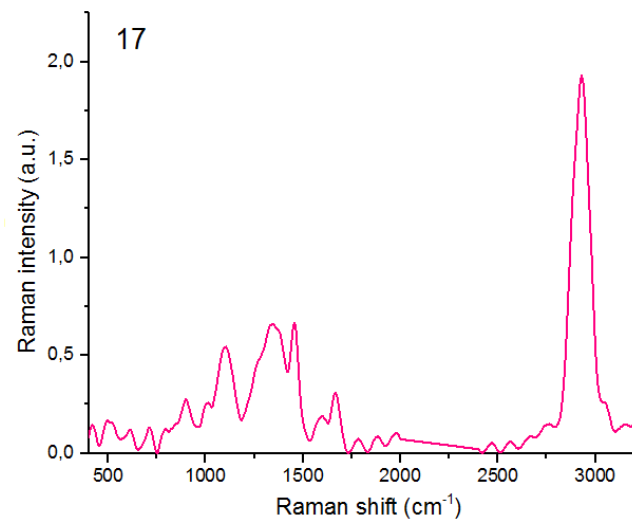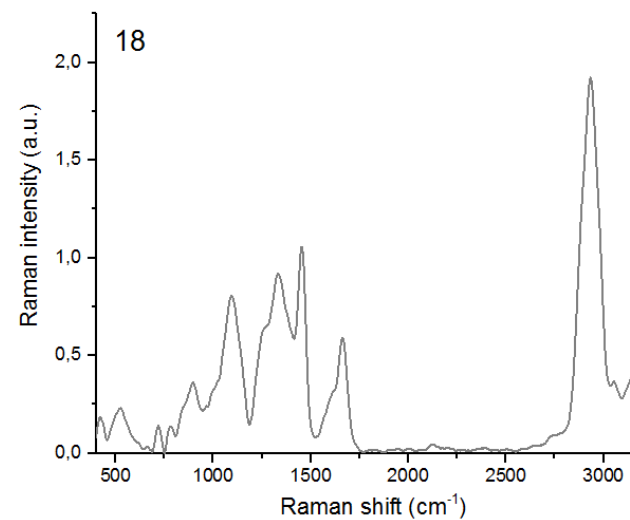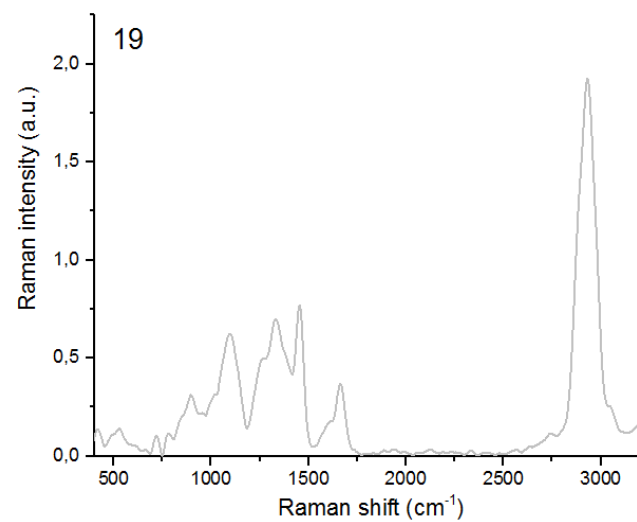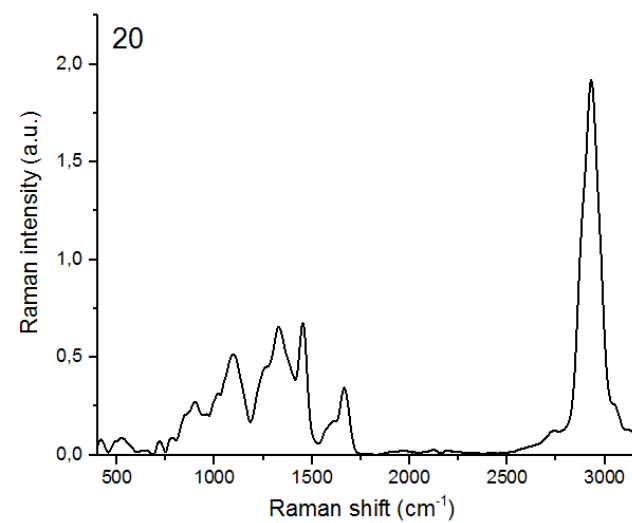

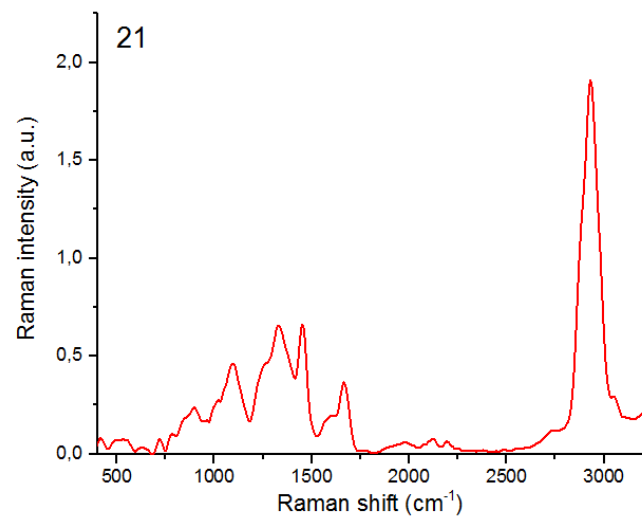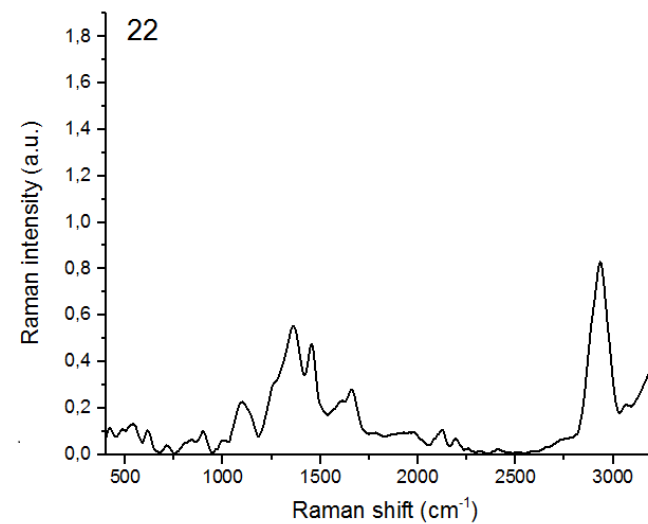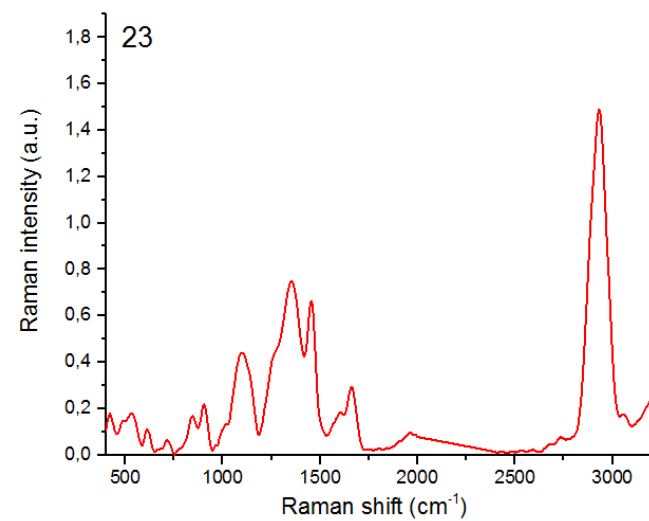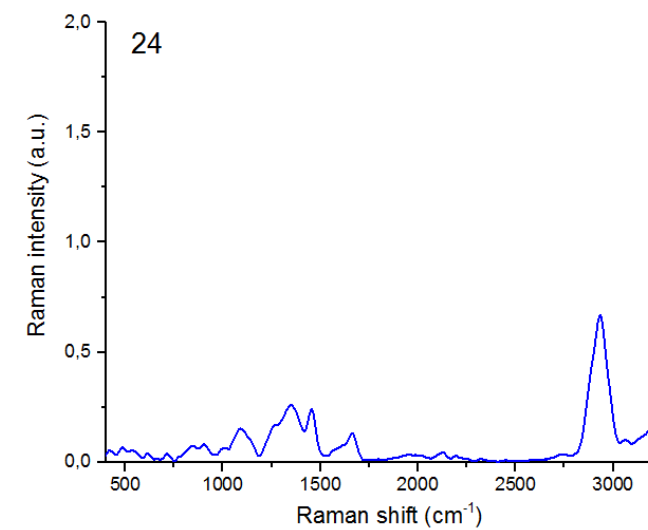

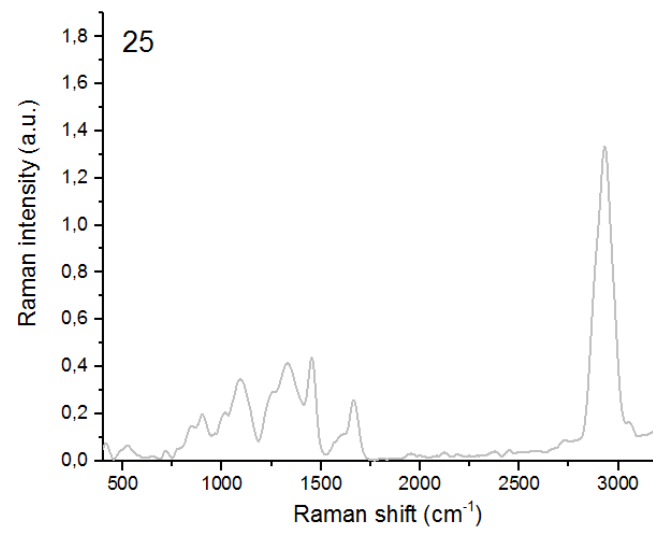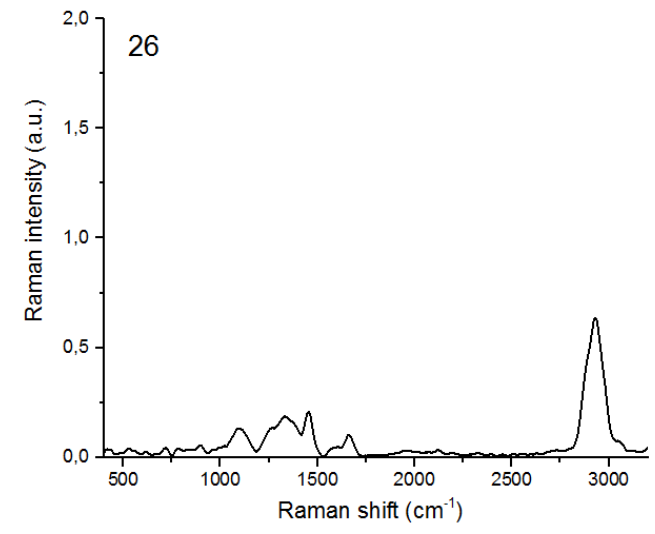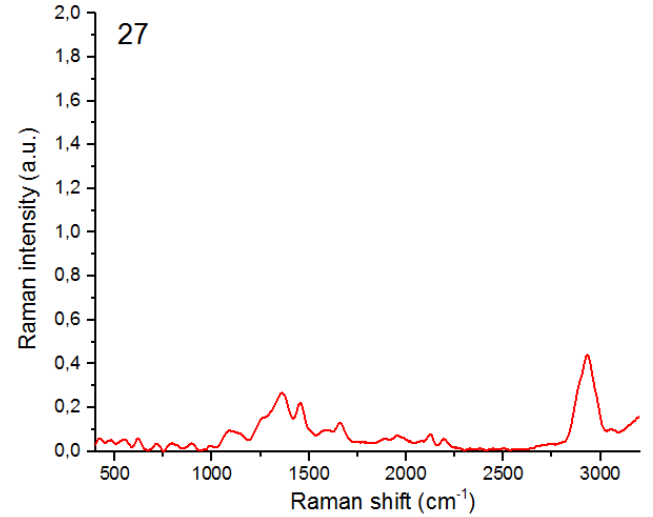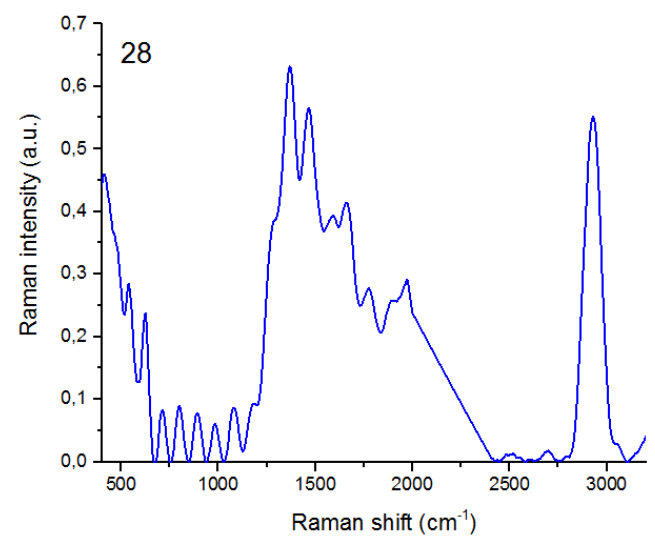

**Supplemental Figure 2.** Raman spectra of clinical *Candida* isolates and reference strains (1-28).

Supplement: Supplementary file 1 [file ijms-20-00988-s001.zip › ijms-439484-supplementary (english edits)/Nowy folder/Supplemental Figure 2.pdf]
