# Supplementary material for: FTIR and Raman Spectroscopy-Based Biochemical Profiling Reflects Genomic Diversity of Clinical Candida Isolates That May Be Useful for Diagnosis and Targeted Therapy of Candidiasis
Source: Int J Mol Sci. 2019 Feb 25;20(4):988. doi: 10.3390/ijms20040988 (PMC6412866; doi:10.3390/ijms20040988)
Supplement: Supplementary file 1 [file ijms-20-00988-s001.zip › ijms-439484-supplementary (english edits)/Nowy folder/Supplemental Table 1 (english edits).doc]

**Supplemental Table 1.** The peak positions and comments on vibrations for clinical *Candida* isolates and reference strains based on both A) FTIR spectroscopy and B) Raman spectroscopy.

| **A) FTIR spectroscopy—peak positions (wavenumber, cm^–1^)** | | | | | | | | | | | | | | | | | | | | | | | | | | | | |
| --- | --- | --- | --- | --- | --- | --- | --- | --- | --- | --- | --- | --- | --- | --- | --- | --- | --- | --- | --- | --- | --- | --- | --- | --- | --- | --- | --- | --- |
| **1** | **2** | **3** | **4** | **5** | **6** | **7** | **8** | **9** | **10** | **11** | **12** | **13** | **14** | **15** | **16** | **17** | **18** | **19** | **20** | **21** | **22** | **23** | **24** | **25** | **26(n)** | **27(2n)** | **28(4n)** | **Vibrations** |
| 879 | 898 | 860 | 881 | 885 | 892 | 881 | 921 | 894 | 914 | 925 | 912 | 915 | 914 | 894 | 883 | 915 | 914 | 914 | 911 | 914 | 885 | 900 | 921 | 908 | 867 | 848 | 904 | C-O, C-O-H, and C-O-C deformation and  C-C stretching vibrations of carbohydrates |
| 1074 | 1054 | 1062 | 1062 | 1058 | 1058 | 1060 | 1043 | 1066 | 1052 | 1064 | 1062 | 1041 | 1076 | 1066 | 1062 | 1031 | 1066 | 1064 | 1066 | 1066 | 1066 | 1060 | 1054 | 1077 | 1072 | 1064 | 1062 | β(1–3) glucans, nucleic acids and glycogen, PO^2-^ symmetric stretching vibrations mainly from RNA |
| 1247 | 1236 | 1239 | 1236 | 1232 | 1230 | 1234 | 1216 | 1230 | 1245 | 1238 | 1241 | 1239 | 1229 | 1238 | 1224 | 1236 | 1224 | 1224 | 1232 | 1228 | 1232 | 1234 | 1230 | 1230 | 1228 | 1245 | 1245 | C-O asymmetric stretching vibrations in phospholipids, lipids |
| 1290 | 1296 | 1311 | 1305 |  | 1309 |  | 1319 | 1303 | 1303 | 1299 | 1303 | 1309 | 1311 | 1313 | 1297 | 1313 | 1299 | 1297 | 1315 | 1301 | 1292 | 1292 | 1301 | 1307 | 1297 | 1311 | 1297 | Amide III: C–N and C–O stretching vibrations, N–H and O=C–N bending vibrations |
| 1398 | 1396 | 1392 | 1392 | 1405 | 1390 | 1392 | 1407 | 1413 | 1398 | 1399 | 1394 | 1404 | 1396 | 1394 | 1390 | 1386 | 1411 | 1394 | 1394 | 1411 | 1409 | 1392 | 1392 | 1394 | 1407 | 1392 | 1386 | C=O of COO^-^ symmetric stretching vibrations in proteins, CH_2_ wagging vibrations in lipids and β(1–3) glucans |
| 1540 | 1544 | 1531 | 1548 | 1538 | 1544 | 1535 | 1540 | 1538 | 1538 | 1536 | 1550 | 1550 | 1550 | 1536 | 1538 | 1536 | 1536 | 1538 | 1538 | 1538 | 1552 | 1538 | 1538 | 1536 | 1550 | 1550 | 1538 | Amide II: mainly C–N stretching vibrations and N–H bending vibrations |
| 1616 | 1633 | 1637 | 1631 | 1629 | 1631 | 1623 | 1643 | 1643 | 1633 | 1629 | 1643 | 1627 | 1627 | 1643 | 1639 | 1631 | 1643 | 1635 | 1644 | 1631 | 1648 | 1633 | 1633 | 1648 | 1621 | 1637 | 1621 | Amide I: mainly C=O stretching vibrations and contributions of N–H bending vibrations |
| 1735 | 1734 | 1733 | 1729 | 1724 | 1727 | 1708 | 1766 |  |  | 1787 | 1747 | 1724 |  | 1776 | 1733 | 1783 | 1770 |  |  | 1718 | 1741 | 1724 |  | 1724 | 1718 | 1724 | 1702 | C=O stretching vibrations in lipid esters |
| 2914 | 2902 | 2909 | 2896 | 2894 | 2911 | 2894 | 2848 | 2861 | 2846 | 2842 | 2852 | 2850 | 2865 | 2864 | 2925 | 2909 | 2917 | 2848 | 2865 | 2850 |  | 2855 |  |  | 2892 | 2898 | 2871 | CH_2_ stretching vibrations in lipids |
| 2973 | 2962 | 2967 | 2969 | 2973 | 2975 | 2985 | 2935 | 2933 | 2921 | 2915 | 2917 | 2927 | 2937 | 2933 | 2948 | 2933 | 2957 | 2936 | 2935 | 2921 | 2919 | 2921 | 2921 | 2923 | 2969 | 2963 | 2929 | CH_3_ stretching vibrations in lipids |
| 3257 | 3264 | 3264 | 3262 | 3237 | 3266 | 3237 | 3243 | 3268 | 3253 | 3253 | 3274 | 3259 | 3337 | 3268 | 3232 | 3251 | 3259 | 3268 | 3274 | 3243 | 3264 | 3249 | 3276 | 3268 | 3249 | 3237 | 3239 | OH from water |
| 3397 | 3407 | 3401 | 3394 | 3378 | 3394 | 3374 | 3368 | 3395 | 3389 | 3388 | 3380 | 3353 | 3401 | 3365 | 3386 | 3361 | 3365 | 3390 | 3417 | 3409 | 3411 | 3417 | 3428 | 3436 | 3380 |  | 3367 | Amide A from proteins |
| **B) Raman spectroscopy—peak positions (Raman shift, cm^–1^)** | | | | | | | | | | | | | | | | | | | | | | | | | | | | |
| **1** | **2** | **3** | **4** | **5** | **6** | **7** | **8** | **9** | **10** | **11** | **12** | **13** | **14** | **15** | **16** | **17** | **18** | **19** | **20** | **21** | **22** | **23** | **24** | **25** | **26(n)** | **27(2n)** | **28(4n)** | **Vibrations** |
| 904 | 912 | 915 | 912 | 888 | 912 | 904 | 888 | 881 | 885 | 881 | 885 | 881 | 885 | 885 | 888 | 888 | 908 | 908 | 888 | 885 | 908 | 915 | 896 | 912 | 888 | 885 | 881 | symmetric benzene/  pyrrole in-phase and out of phase breathing mode of  tryptophan and phenylalanine |
| 981 | 1016 | 1035 | 1020 | 1031 | 1077 | 1008 | 1020 | 1000 | 1004 | 1004 | 1008 | 1016 | 1008 | 1008 | 1008 | 996 | 1020 | 1035 | 1035 | 1004 | 1000 | 1008 | 1012 | 1004 | 1016 | 981 | 993 | symmetric benzene/  pyrrole in-phase and out of phase breathing mode of  tryptophan and phenylalanine |
| 1317 | 1320 | 1320 | 1320 | 1320 | 1340 | 1317 | 1317 | 1363 | 1320 | 1359 | 1320 | 1347 | 1344 | 1317 | 1344 | 1371 | 1320 | 1317 | 1344 | 1344 | 1344 | 1340 | 1332 | 1320 | 1320 | 1367 | 1359 | C-H deformation vibrations from proteins |
| 1459 | 1459 | 1444 | 1444 | 1463 | 1444 | 1444 | 1444 | 1440 | 1444 | 1440 | 1444 | 1463 | 1440 | 1444 | 1444 | 1444 | 1463 | 1463 | 1463 | 1440 | 1463 | 1448 | 1444 | 1444 | 1444 | 1459 | 1471 | C-H deformation vibrations from lipids |
| 1587 | 1598 | 1579 | 1602 | 1587 | 1594 | 1590 | 1606 | 1579 | 1587 | 1575 | 1590 | 1579 | 1598 | 1606 | 1556 | 1579 | 1583 | 1594 | 1579 | 1594 | 1583 | 1579 | 1575 | 1598 | 1567 | 1598 | 1575 | ring stretching vibrations of the deoxyribonucleotides adenosine  monophosphate |
| 1648 | 1671 | 1648 | 1652 | 1671 | 1675 | 1671 | 1648 | 1671 | 1656 | 1675 | 1675 | 1652 | 1652 | 1671 | 1648 | 1652 | 1652 | 1668 | 1652 | 1652 | 1648 | 1671 | 1656 | 1652 | 1652 | 1652 | 1668 | Amide I: mainly C=O stretching vibrations and contributions of N–H bending vibrations |
| 2929 | 2940 | 2940 | 2944 | 2917 | 2940 | 2944 | 2921 | 2921 | 2944 | 2940 | 2917 | 2944 | 2940 | 2944 | 2944 | 2940 | 2917 | 2944 | 2917 | 2944 | 2921 | 2917 | 2944 | 2917 | 2913 | 2948 | 2917 | C-H stretching vibration from lipids |

As we have classified clinical *Candida* isolates into three species (Table 1), four groups of yeast were considered, namely *C. albicans* isolates (n = 18, samples 1, 2, and from 4 to 19, marked in blue), *C. tropicalis* isolates (n = 2, samples from 20 to 21, marked in red), *C. glabrata* isolates (n = 5, sample 3 and samples from 22 to 25, marked in yellow) and reference strains (n = 3, haploid, diploid, and tetraploid strains, samples from 26 to 28, marked in violet) (Table 2). The shifts in peaks provide information on the structural changes in functional groups of chemical compounds, as well as the changes in the chemical composition. Some peaks are not present in selected samples. In *C. albicans* group (samples 9, 10, 14, 19), a peak corresponding to C=O stretching vibrations in lipid esters is not present. This peak is also not observed in sample 20 (*C. tropicalis* group) and in sample 24 (*C. glabrata* group). The amide III vibrations in samples 5 and 7 (*C. albicans* group) are not present. Moreover, the CH_2_ stretching vibrations in lipids at 2914 cm^–1^ is observed within *C. glabrata* group in sample 23 only. In the FTIR spectrum of sample 27 (2n reference strain), a vibration at 3397 cm^-1^ is not present. However, in Raman spectra of all analyzed samples, the same vibrations are observed. This discrepancy may be due to the fact that FTIR spectroscopy is based on the change of the dipole moment, whereas Raman spectroscopy is based on the change of bond polarity (Matthaus et al., 2008).

Matthaus, C., et al., 2008. Chapter 10: Infrared and Raman microscopy in cell biology. Methods Cell Biol. 89**,** 275–308.
