# Supplementary material for: FTIR and Raman Spectroscopy-Based Biochemical Profiling Reflects Genomic Diversity of Clinical Candida Isolates That May Be Useful for Diagnosis and Targeted Therapy of Candidiasis
Source: Int J Mol Sci. 2019 Feb 25;20(4):988. doi: 10.3390/ijms20040988 (PMC6412866; doi:10.3390/ijms20040988)
Supplement: Supplementary file 1 [file ijms-20-00988-s001.zip › ijms-439484-supplementary (english edits)/Nowy folder/Supplemental Table 2 (english edits).doc]

**Supplemental Table 2.** Analysis of secondary protein structures of clinical *Candida* isolates based on the IR region between 1700–1600 cm^–1^ (amide I region).

| **Peak area** | | | **Percentage [%]** | | | **α-helix /β-sheet ratio** | |  |
| --- | --- | --- | --- | --- | --- | --- | --- | --- |
| **Sample** | **α-helix** | **β-sheet** | | **α-helix** | **β-sheet** | |  | |
| **1** | 11.76 | 11.97 | | 49.56 | 50.44 | | 0.98 | |
| **2** | 14.65 | 12.10 | | 54.77 | 45.23 | | 1.21 | |
| **3** | 13.96 | 11.49 | | 54.85 | 45.15 | | 1.21 | |
| **4** | 10.13 | 9.78 | | 50.88 | 49.12 | | 1.04 | |
| **5** | 8.14 | 8.87 | | 47.85 | 52.16 | | 0.92 | |
| **6** | 9.65 | 9.62 | | 50.08 | 49.92 | | 1.00 | |
| **7** | 6.34 | 7.26 | | 46.62 | 53.38 | | 0.87 | |
| **8** | 21.62 | 17.84 | | 54.79 | 45.21 | | 1.21 | |
| **9** | 8.80 | 30.97 | | 22.13 | 77.87 | | 0.28 | |
| **10** | 15.69 | 23.87 | | 39.66 | 60.34 | | 0.66 | |
| **11** | 19.54 | 26.52 | | 42.42 | 57.578 | | 0.74 | |
| **12** | 21.55 | 17.07 | | 55.80 | 44.20 | | 1.26 | |
| **13** | 18.64 | 19.98 | | 48.27 | 51.73 | | 0.93 | |
| **14** | 19.7 | 17.80 | | 52.53 | 47.47 | | 1.11 | |
| **15** | 23.50 | 17.54 | | 57.26 | 42.74 | | 1.34 | |
| **16** | 18.84 | 13.56 | | 58.15 | 41.85 | | 1.40 | |
| **17** | 12.24 | 13.47 | | 47.61 | 52.39 | | 0.91 | |
| **18** | 20.26 | 14.27 | | 58.67 | 41.33 | | 1.42 | |
| **19** | 14.27 | 16.29 | | 46.70 | 53.30 | | 0.88 | |
| **20** | 37.31 | 17.37 | | 68.23 | 31.77 | | 2.15 | |
| **21** | 41.50 | 21.68 | | 65.69 | 34.31 | | 1.91 | |
| **22** | 28.33 | 15.09 | | 65.25 | 34.75 | | 1.88 | |
| **23** | 22.25 | 19.75 | | 52.98 | 47.02 | | 1.13 | |
| **24** | 35.33 | 21.64 | | 62.02 | 37.98 | | 1.63 | |
| **25** | 23.59 | 45.99 | | 33.90 | 66.10 | | 0.51 | |
| **26** | 18.66 | 4.13 | | 81.88 | 18.12 | | 4.52 | |
| **27** | 19.4 | 12.46 | | 60.89 | 39.11 | | 1.56 | |
| **28** | 23.27 | 13.39 | | 63.46 | 36.52 | | 1.74 | |
