# Supplementary material for: FTIR and Raman Spectroscopy-Based Biochemical Profiling Reflects Genomic Diversity of Clinical Candida Isolates That May Be Useful for Diagnosis and Targeted Therapy of Candidiasis
Source: Int J Mol Sci. 2019 Feb 25;20(4):988. doi: 10.3390/ijms20040988 (PMC6412866; doi:10.3390/ijms20040988)
Supplement: Supplementary file 1 [file ijms-20-00988-s001.zip › ijms-439484-supplementary (english edits)/Nowy folder/Supplemental Table 3.doc]

**Supplemental Table 3.** Lipid-carbohydrate ratio based on FTIR spectroscopy.

| **Lipid-carbohydrate ratio - FTIR spectroscopy** | | | | | | | | | | | | | |
| --- | --- | --- | --- | --- | --- | --- | --- | --- | --- | --- | --- | --- | --- |
| **1** | **2** | **3** | **4** | **5** | **6** | **7** | **8** | **9** | **10** | **11** | **12** | **13** | **14** |
| 0.68 | 0.93 | 0.91 | 0.85 | 0.80 | 0.70 | 0.90 | 0.37 | 0.28 | 0.37 | 0.19 | 0.22 | 0.16 | 0.30 |
| **15** | **16** | **17** | **18** | **19** | **20** | **21** | **22** | **23** | **24** | **25** | **26(n)** | **27(2n)** | **28(4n)** |
| 0.28 | 0.43 | 0.12 | 0.35 | 0.21 | 0.19 | 0.20 | 0.36 | 0.33 | 0.31 | 0.19 | 0.67 | 0.10 | 0.17 |
