# Supplementary material for: FTIR and Raman Spectroscopy-Based Biochemical Profiling Reflects Genomic Diversity of Clinical Candida Isolates That May Be Useful for Diagnosis and Targeted Therapy of Candidiasis
Source: Int J Mol Sci. 2019 Feb 25;20(4):988. doi: 10.3390/ijms20040988 (PMC6412866; doi:10.3390/ijms20040988)
Supplement: Supplementary file 1 [file ijms-20-00988-s001.zip › ijms-439484-supplementary (english edits)/Nowy folder/Supplemental Table 4 (English edits).doc]

| **FTIR spectroscopy –peak area for lipid-carbohydrate ratio** | | | | | | | | | | | | | | | | | | | | | | | | | | | | |
| --- | --- | --- | --- | --- | --- | --- | --- | --- | --- | --- | --- | --- | --- | --- | --- | --- | --- | --- | --- | --- | --- | --- | --- | --- | --- | --- | --- | --- |
| **1** | **2** | **3** | **4** | **5** | **6** | **7** | **8** | **9** | **10** | **11** | **12** | **13** | **14** | **15** | **16** | **17** | **18** | **19** | **20** | **21** | **22** | **23** | **24** | **25** | **26(n)** | **27(2n)** | **28(4n)** |  |
| **879** | **898** | **860** | **881** | **885** | **892** | **881** | **921** | **894** | **914** | **925** | **912** | **915** | **914** | **894** | **883** | **915** | **914** | **914** | **911** | **914** | **885** | **900** | **921** | **908** | **867** | **848** | **904** | Peak |
| 2.98 | 4.62 | 3.59 | 4.75 | 4.74 | 4.11 | 4.90 | 1.17 | 2.74 | 2.24 | 5.70 | 2.76 | 3.21 | 4.41 | 1.88 | 8.64 | 0.73 | 2.18 | 0.34 | 2.40 | 4.45 | 4.88 | 5.39 | 5.78 | 3.25 | 11.18 | 10.74 | 13.70 | Peak area |
| **1074** | **1054** | **1062** | **1062** | **1058** | **1058** | **1060** | **1043** | **1066** | **1052** | **1064** | **1062** | **1041** | **1076** | **1066** | **1062** | **1031** | **1066** | **1064** | **1066** | **1066** | **1066** | **1060** | **1054** | **1077** | **1072** | **1064** | **1062** | Peak |
| 122.94 | 100.61 | 137.30 | 118.48 | 99.73 | 120.45 | 87.55 | 160.61 | 160.90 | 159.90 | 175.15 | 167.28 | 176.04 | 150.08 | 157.89 | 165.49 | 116.12 | 153.25 | 145.58 | 188.38 | 179.76 | 189.31 | 211.37 | 212.87 | 195.75 | 153.95 | 159.21 | 154.21 | Peak area |
| **1735** | **1734** | **1733** | **1729** | **1724** | **1727** | **1708** | **1766** |  |  | **1787** | **1747** | **1724** |  | **1776** | **1733** | **1783** | **1770** |  |  | **1718** | **1741** | **1724** |  | **1724** | **1718** | **1724** | **1702** | Peak |
| 0.39 | 0.18 | 0.37 | 0.34 | 0.27 | 0.44 | 0.48 | 0.22 | 0 | 0 | 0.15 | 0.18 | 1.16 | 0 | 0.26 | 0.42 | 1.39 | 0.46 | 0 | 0 | 0.70 | 0.74 | 0.55 | 0 | 0.92 | 0.97 | 0.39 | 0.62 | Peak area |
| **2914** | **2902** | **2909** | **2896** | **2894** | **2911** | **2894** | **2848** | **2861** | **2846** | **2842** | **2852** | **2850** | **2865** | **2864** | **2925** | **2909** | **2917** | **2848** | **2865** | **2850** |  | **2855** |  |  | **2892** | **2898** | **2871** | Peak |
| 42.82 | 47.01 | 57.15 | 48.82 | 43.58 | 43.50 | 36.68 | 15.86 | 25.10 | 37.58 | 18.13 | 19.75 | 15.53 | 26.29 | 27.49 | 49.93 | 8.14 | 35.73 | 14.51 | 16.45 | 15.84 | 0 | 12.26 | 0 | 0 | 30.97 | 5.64 | 15.88 | Peak area |
| **2973** | **2962** | **2967** | **2969** | **2973** | **2975** | **2985** | **2935** | **2933** | **2921** | **2915** | **2917** | **2927** | **2937** | **2933** | **2948** | **2933** | **2957** | **2936** | **2935** | **2921** | **2919** | **2921** | **2921** | **2923** | **2969** | **2963** | **2929** | Peak |
| 42.02 | 50.17 | 70.78 | 55.11 | 39.22 | 42.64 | 45.76 | 43.52 | 19.92 | 22.20 | 16.35 | 17.29 | 11.52 | 20.62 | 16.26 | 25.37 | 3.93 | 17.78 | 16.26 | 18.88 | 20.74 | 68.63 | 59.64 | 66.82 | 37.34 | 78.15 | 10.33 | 12.80 | Peak area |

**Supplemental Table 4.** FTIR spectroscopy—peak area for lipid-carbohydrate ratio of clinical *Candida* isolates and reference strains (1–28).
